# Curcumin Mitigates AFB1-Induced Hepatic Toxicity by Triggering Cattle Antioxidant and Anti-inflammatory Pathways: A Whole Transcriptomic In Vitro Study

**DOI:** 10.3390/antiox9111059

**Published:** 2020-10-29

**Authors:** Marianna Pauletto, Mery Giantin, Roberta Tolosi, Irene Bassan, Andrea Barbarossa, Anna Zaghini, Mauro Dacasto

**Affiliations:** 1 Division of Pharmacology and Toxicology, Department of Comparative Biomedicine and Food Science, University of Padova, viale dell’Università 16, Legnaro, 35020 Padova, Italy; marianna.pauletto@unipd.it (M.P.); mery.giantin@unipd.it (M.G.); roberta.tolosi@phd.unipd.it (R.T.); irene.bassan@unipd.it (I.B.); 2 Department of Veterinary Medical Sciences, University of Bologna, Via Tolara di Sopra 50, Ozzano dell’Emilia, 40064 Bologna, Italy; andrea.barbarossa@unibo.it (A.B.); anna.zaghini@unibo.it (A.Z.)

**Keywords:** aflatoxin B1, aflatoxicosis, mycotoxins, curcumin, antioxidants, cattle, liver, RNAseq, transcriptome, cancer

## Abstract

Aflatoxin B1 (AFB1) toxicity in livestock and human beings is a major economic and health concern. Natural polyphenolic substances with antioxidant properties have proven to be effective in ameliorating AFB1-induced toxicity. Here we assessed the potential anti-AFB1 activity of curcumin (pure curcumin, C, and curcumin from *Curcuma longa*, CL) in a bovine fetal hepatocyte-derived cell line (BFH12). First, we measured viability of cells exposed to AFB1 in presence or absence of curcumin treatment. Then, we explored all the transcriptional changes occurring in AFB1-exposed cells cotreated with curcumin. Results demonstrated that curcumin is effective in reducing AFB1-induced toxicity, decreasing cells mortality by approximately 30%. C and CL induced similar transcriptional changes in BFH12 exposed to AFB1, yet C treatment resulted in a larger number of significant genes compared to CL. The mitigating effects of curcuminoids towards AFB1 toxicity were mainly related to molecular pathways associated with antioxidant and anti-inflammatory response, cancer, and drug metabolism. Investigating mRNA changes induced by curcumin in cattle BFH12 cells exposed to AFB1 will help us to better characterize possible tools to reduce its consequences in this susceptible and economically important food-producing species.

## 1. Introduction

Aflatoxins (AFs) are secondary metabolites mainly produced by *Aspergillus flavus* and *Aspergillus parasiticus* [1]. They are difuranocoumarin derivatives comprising aflatoxin B1, B2, G1, G2, M1, and M2. Aflatoxins are known to affect important agricultural feed and food commodities (e.g., dried fruits, oilseed meals, spices, and cereals), causing various diseases and health issues in humans and farmed animals. Aflatoxin B1 (AFB1) consumption has been strongly associated with human hepatocellular carcinoma (HCC), and the International Agency for Research on Cancer (IARC) has classified this mycotoxin as a Group I agent [2].

Aflatoxins prevalence in animal feed is particularly significant in developing countries close to the equator [3]. Nevertheless, due to increased average temperature, AFB1 has become a wider concern affecting also geographical areas in South Eastern Europe [4]. Exposure to AFB1 through contaminated food ingredients might have severe implications in farmed animals, such as liver damage, nutritional or immunological consequences. These effects are associated to decreased growth and productivity [5,6,7,8], ultimately leading to significant economic losses [9,10].

Aflatoxin B1 is a “pro-carcinogen” and its toxicity is associated to bioactivation and detoxification pathways. In particular, AFB1 is biologically activated by cytochrome P450 (CYP) 1A1 (a member of the CYP superfamily of phase I drug metabolizing enzymes) to an extremely reactive and electrophilic derivative, the AFB1-8,9-epoxide (AFBO). The aflatoxin epoxide covalently binds to macromolecules such as DNA and proteins, thereby forming adducts, which are responsible for severe toxicity and eventually of carcinogenic activity [11]. The detoxification of AFBO mainly occurs via its conjugation with glutathione (GSH) by glutathione S-transferase (GST), a phase II conjugative (detoxification) enzyme [11]. Notably, besides AFBO, the animal metabolism can also produce relatively less toxic metabolites, such as aflatoxicol (AFL) or aflatoxin M1 (AFM1) [11]. Remarkably, species-differences in the constitutive expression and catalytic activity of these metabolic pathways and enzymes appear responsible for different susceptibility to AFB1 among farmed animals [12].

Cattle are relatively more resistant to AFs mainly because of the presence of rumen microflora, which is able to convert part of the ingested AFB1 into less toxic or nontoxic metabolites [13]. However, AFs interfere with the overall ruminal digestive capacity [14], and are absorbed at intestinal level. Besides animal symptoms (e.g., impaired reproductive efficiency, diarrhea, mastitis), affecting growth performance, AFB1 in dairy cattle is also a public health concern as it might lead to AFM1 milk carryover [15].

Since avoiding all the potential exposure to AFs is not feasible, chemical (e.g., clay-based adsorbents, vitamins A, C, and E) and biological (e.g., bacteria, botanical extracts) feed additives have been extensively examined for their ability to prevent or mitigate aflatoxicosis in farmed animals (e.g., [9,16,17,18,19,20,21]). Among the botanical feed additives with a potential anti-AFB1 activity, curcumin is one of the most attractive [22].

Curcumin is a polyphenolic carotenoid isolated from turmeric (*Curcuma longa*). From a chemical point of view, turmeric is a mixture of water-insoluble curcuminoids (i.e., curcumin, demethoxycurcumin, bisdemethoxycurcumin), a water-soluble peptide (turmerin), and essential oils (turmerones, altertones, zingiberene) [23]. Curcuminoids constitute a variable part (i.e., 2–9% of dry weight) of turmeric, and curcumin makes up to 60–70% of total curcuminoids extract [24]. Curcumin has a variety of pharmacological activities: antioxidant, anti-inflammatory, chemopreventive, antimicrobial, antiviral, and antifungal properties have been broadly reported in humans and rodents [25].

So far, several studies have demonstrated the potential of curcumin, both in vivo and in vitro, in ameliorating cancer-related damage and carcinogenesis (e.g., [26,27,28,29]) and improving antioxidant status (e.g., [30,31,32,33]). Curcumin was found to inhibit metabolic transformation of AFs to their hepatotoxic or carcinogenic derivatives in chicken liver cytosol [34]. In rats, curcumin contributes to maintain the antioxidant status in hepatic cells by scavenging free radicals, inhibiting oxidative enzymes, activating catalases, and inducing de novo glutathione synthesis, thus having protective effects against AFB1-induced liver injury [30]. Likewise, a transcriptional upregulation of the antioxidant enzymes GST, superoxide dismutase (SOD), catalase (CAT), and glutathione peroxidase (GPX) was reported in rat liver upon oral curcumin and AFB1 cotreatment [31].

Curcumin has also shown to affect mRNA and the activity of drug metabolizing enzymes involved in AFB1 metabolism and detoxification. For instance, mRNA levels and/or enzyme activities of CYP1A1, CYP1A2, CYP2A6, and CYP3A4 isoenzymes were increased upon AFB1 exposure in broilers, while they were inhibited when curcumin was used as feed supplementation [35]. Similarly, curcumin selectively inhibited the hepatic CYP2A6 expression in broilers treated with AFB1 and curcumin supplementation [36]. In broilers fed AFB1 (5 mg/kg feed) and curcumin (450 mg/kg feed), the expression level of the nuclear factor erythroid 2-related factor 2 (NRF2), and its downstream genes such as GSTA3 and GSTM2, was significantly upregulated compared to animals fed only with AFB1 [37]. Moreover, feed supplementation with curcumin exhibited protective effects on AFB1-induced liver toxicity in Nile tilapia by maintaining the regular expression of CYP1A, SOD, interleukin 1β (IL1β), and transforming growth factor beta (TGFβ) [38].

Despite the benefits of curcumin in tackling aflatoxicosis and, more in general, any other oxidative stress condition, a limited number of studies have investigated in depth the molecular mechanisms underlying protective mechanisms. Regarding farmed animals, the only studies published so far are limited to poultry species [33,36,39,40,41,42], except for an in vitro approach investigating how curcumin modulates AFB1 toxicity in a bovine mammary epithelial cell line [20]. To date, no studies assessing the potential protective role of curcuminoids on AFB1-mediated whole transcriptional changes in an established cell line isolated from veterinary species have ever been published.

A previously published study demonstrated that AFB1 has detrimental effects on a bovine fetal hepatocyte-derived cell line (BFH12) [43] that constitutively expresses most of cattle drug metabolizing enzymes and transporters [44,45]; moreover, the authors proved that AFB1 deeply impacts the whole transcriptome of BFH12 cells. Based on these considerations, the aim of the present study was to assess the protective role of curcuminoids in BFH12 cells exposed to AFB1, and to elucidate the whole transcriptional changes (RNA-seq) triggered by this natural polyphenolic substance when administered in combination with AFB1. To reach such a goal, cells were at first pretreated for 16 h with either pure curcumin or a mix of curcuminoids from *C. longa* (including curcumin); then, monolayers were exposed to 3.6 μM AFB1 for further 48 h in combination with the antioxidants cited above. As a control, cells exposed to curcumin or curcuminoids were also included in the experiment. The outcomes of this study might help in developing curcuminoid-supplemented diets to mitigate AFB1 effects in dairy cattle, thus avoiding significant economic losses and reducing the risk of dairy products contamination.

## 2. Materials and Methods

### 2.1. Materials

Cell culture flasks and multi-well plates were purchased from Sarstedt (Verona, Italy). Williams’ Medium E, L-alanyl-l-glutamine, penicillin/streptomycin, and fetal bovine serum (FBS) were acquired from Biochrom (Biospa, Milan, Italy).

Aflatoxin B1, curcumin (≥94% purity, ≥80% curcumin, C), curcumin from *Curcuma longa* (≥65%, purity, CL), and dimethyl sulfoxide (DMSO) were obtained from Sigma-Aldrich (St. Louis, MO, USA). Polychlorinated biphenyl 126 (99%, PCB126) and aflatoxin M1 (AFM1) were purchased from Lab Service analytica (Bologna, Italy), aflatoxicol from DBA Italia (Milano, Italy), and 13C17-Aflatoxin B1 from Orsell (Modena, Italy). All other chemicals used in the study are commercially available and of molecular biology grade. Solvents used for metabolites quantification were all of LC-MS grade.

### 2.2. Cell Culture

The bovine SV40 large T-antigen-transduced fetal hepatocyte-derived cell line BFH12 [44] was kindly provided by Dr. Axel Schoeniger (Institute of Biochemistry, University of Leipzig, Leipzig, Germany). The general conditions of cell line maintenance have been described in [43].

### 2.3. Curcuminoids Cytotoxicity

Cells were seeded in 96-well flat bottom plates at a density of 6 × 10^3^ cells/well. Four days after seeding, cells were exposed to increasing concentrations of C or CL for a total of 64 h (range 10–40 μM). Curcuminoids were dissolved in DMSO (*v*:*v*), whose final concentration never exceeded 0.1%; additional wells exposed to either the vehicle or to cell medium were prepared, too. Cell viability was measured using WST-1 Cell Proliferation Reagent (Roche, Basel, Switzerland) as previously described [43] and expressed as the percentage relative to that of cells exposed to the vehicle only (0.1% DMSO). Experiments were performed at least in triplicate, and each concentration was tested in sextuplicate.

Afterwards, we assessed the ability of different concentrations of C and CL to reduce AFB1-induced cytotoxicity [43]. Assuming the metabolic competence of fetal hepatocytes to be lower compared to that of adult liver cells, also in this case we opted for a treatment with an AHR agonist, i.e., PCB126, thus increasing the responsiveness to AFB1. Cells in monolayer were pretreated with PCB126 (1 nM) for 24 h; the PCB126 treatment was stopped when changing the medium; after a 8 h rest, cells were exposed to C or CL (2.5, 5, and 10 μM) for 16 h; finally, cells were cotreated with C or CL and AFB1 (3.6 μM) for further 48 h. At the end of each incubation, cytotoxicity was assessed using the WST-1 Cell Proliferation Reagent (Roche). The cell viability was expressed as the percentage relative to that of control cells exposed to PCB126.

The AFB1 and PCB126 concentrations employed in this study were selected based on previously published results [43]; as to C and CL, concentrations were chosen based on the half maximal inhibitory concentration (IC_50_) obtained in the present study. The selected concentrations were always below the corresponding IC_50_.

All the incubations with the mycotoxin were performed using media without FBS, to avoid the possible binding of a considerable proportion of AFB1 to serum albumin [46].

### 2.4. Cells Incubation for Gene Expression Analysis

To measure the effects of curcuminoids (C and CL) treatment upon AFB1-induced transcriptional changes (qPCR and RNA-seq), four independent cell culture experiments were set up. Briefly, BFH12 cells were seeded in 6-well culture plates at a density of 5 × 10^4^ cells/well and exposed to PCB126, C, or CL and AFB1 following the same treatment scheme reported above for cytotoxicity evaluation. Cells pretreated with PCB126 for 24 h and exposed to the vehicle (0.1% DMSO) until the end of the experiment were used as control. Notably, such experimental approach is in accordance with several studies investigating the protective effects of antioxidants against oxidative stress in vitro [20,47,48,49].

At the end of the incubation time, the medium was removed, cells were collected and subjected to total RNA extraction as previously reported [43]. Quali- and quantitative parameters of the nucleic acid were assessed as described in [43]. All samples had an RNA Integrity Number (RIN) value > 7.

### 2.5. Quantitative Real-Time PCR (qPCR)

A targeted qPCR approach was preliminarily performed to assess the effects of C and CL at the concentrations selected on the basis of the dose-response curves (i.e., 2.5, 5, and 10 μM). Only transcripts known to be regulated by AFB1 (i.e., genes involved in AFB1 metabolism and antioxidant response) were considered.

The target gene expression analysis was carried out on four biological replicates (i.e., independent cell culture experiments). A total of 14 experimental conditions were assessed: CTRL, C (2.5, 5, and 10 μM), CL (2.5, 5, and 10 μM), AFB1, C (2.5, 5, and 10 μM) + AFB1, CL (2.5, 5, and 10 μM) + AFB1. Reverse transcription and qPCR amplification conditions were described elsewhere [43]. The whole list of target genes and primers used for qPCR analyses are reported in Table S1. Messenger RNA relative quantification (RQ) was performed using the ΔΔCt method [50] and the Ribosomal Protein Lateral Stalk Subunit P0 (RPLP 0) and Tata Binding Protein (TBP) were used as reference genes. Data are expressed as fold change of treated versus control cells (i.e., treated with PCB126) ± mean standard error (SEM).

### 2.6. RNA-Seq Library Preparation and Sequencing

**Figure 1:**
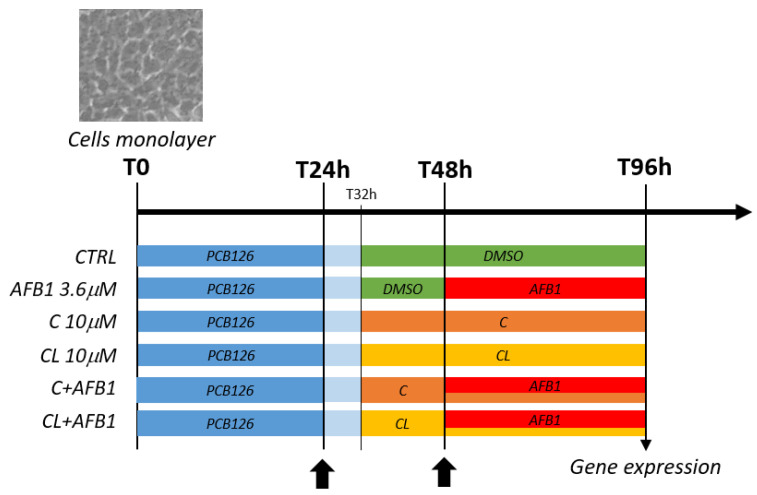
Summary of cell treatments. Scheme reporting the cell treatments performed in this study and the resulting experimental groups assayed with the RNA-seq approach. C = curcumin; CL = *Curcuma longa*. Arrows indicate complete medium changes (fresh medium and treatment solutions).

As far as the RNA-seq analysis is concerned, based on the preliminary qPCR results, only cells exposed to the maximum concentration of C and CL (i.e., 10 μM) were taken into consideration (Figure 1).

For each experimental condition (i.e., CTRL, C 10 μM, CL 10 μM, AFB1, C 10 μM + AFB1, CL 10 μM + AFB1), three independent biological replicates (i.e., independent cell culture experiments) were executed. A total of 18 tagged RNA-seq libraries were prepared and sequenced in an Illumina Hi-Seq 4000 instrument, as previously reported in [43].

Notably, libraries representing CTRL and AFB1 conditions (i.e., 6 libraries) have been previously analyzed in a stand-alone study assessing the whole transcriptional effects of PCB126 and AFB1 on BFH12 cells [43]. In the present study, these libraries were normalized and analyzed again in the context of a larger dataset including new data (i.e., cells treated with C and CL).

### 2.7. Differential Expression Analysis

Reads that passed the quality control check, were trimmed, filtered out, and mapped against the reference *Bos taurus* genome as previously reported [43]. Analysis of differential gene expression was then conducted in EdgeR [51]. Samples were grouped according to treatments (i.e., CTRL, C, CL, AFB1, C + AFB1, CL + AFB1) and pairwise analyses were performed to assess the transcriptional changes induced by curcuminoids alone (i.e., C vs. CTRL, CL vs. CTRL) and curcuminoids in combination with AFB1 (i.e., C + AFB1 vs. AFB1, CL + AFB1 vs. AFB1). Genes showing an extremely low expression level in all samples were filtered out by using the *filterByExpr* function, and extracted reads were normalized with the trimmed mean of M-values (TMM) method. After estimating common and tag-wise dispersions (*estimateDisp*), and fitting a linear model (*glmQLFit*), the function *glmTREAT* was used to find out the differential expressed genes (DEGs), with a FDR set at ≤0.05 and a threshold for a significant Fold Change (FC) set at ≥1.5. The complete R code used for the differential expression analysis is reported in File S1.

### 2.8. Functional Enrichment Analysis

A functional interpretation of significant DEGs was obtained through Gene Ontology (GO) and KEGG over-representation tests (*enrichGO* and *enrichKEGG*), carried out in R environment using the *ClusterProfiler* package [52]. Ensembl gene identifiers were used to establish two different gene lists (i.e., significantly up- and downregulated genes) and a “background” (i.e., all the expressed genes). Using functions included in the *ClusterProfiler* package, dotplots and gene-concept networks were also constructed. Before creating dotplots and gene-concept networks, GO terms redundancy was removed by using the *simplify* function (similarity cutoff = 0.5). Dotplots display the most significant enriched terms (*p*-value ≤ 0.05), while gene-concept networks highlight which genes were involved in the significant GO terms. The detailed R code used for this analysis is reported in File S1.

A pre-ranked KEGG Gene Set Enrichment Analysis (GSEA) [53] was performed to determine whether gene sets defined a priori show statistically significant enrichment at either end of the ranking. A statistically significant enrichment value (Benjamini–Hochberg adjusted *p* value ≤ 0.05) indicates that the biological activity (e.g., the biomolecular pathway), characterized by the gene set, is correlated with the supplied ranking. The input was prepared as follows: the raw *p* values obtained through the DE analysis (i.e., EdgeR, C + AFB1 vs. AFB1) were used to rank the list of genes by significance. When multiple genes with the same gene name were detected, only the most significant one (based on *p* value) was retained. To specify the direction of the gene expression variation, the *p* values (pval) were replaced by 1-pval or -(1-pval) when a gene was overexpressed or underexpressed in the “C + AFB1” group, respectively. The analysis was carried out by using the *gseKEGG* function provided by the *ClusterProfiler* package [52]. The detailed R code used for this analysis is reported in File S1.

### 2.9. Analytical Investigations

At the end of the experiment (T96 h), total AFB1, AFM1, and AFL were measured by LC-MS/MS in all the experimental conditions, as previously specified [43]. Both medium and cells samples were considered.

### 2.10. Oxidative Stress

To confirm the pro-oxidant effects of AFB1 and assess the potential benefits of curcuminoids, we measured lipid peroxidation (malondialdehyde production) in all the experimental groups. Briefly, BFH12 cells were seeded in standard 90 mm Petri dishes at a density of 3 × 10^5^ cells/plate and treated using the same protocol (time of incubation and concentrations) used for RNA-seq experiments (see Figure 1). Six independent biological replicates (i.e., independent cell culture experiments) were executed. At the end of the incubation time, the medium was removed, cells were washed twice and scraped off with 500 µL of ice-cold Tris HCl 20 mM. Cells suspensions were then mechanically lysed (freezing in liquid nitrogen and immediate thawing at 37 °C for three times, followed by disruption with a steel pestle) and centrifuged at 4 °C for 10 min at 12,000 rpm. The supernatant was finally collected and stored on ice until use. Total protein content was measured using the BCA assay kit (Life Technologies, Carlsbad, CA, USA). The amount of malondialdehyde (MDA) was quantified in undiluted cell lysates using the ab233471 Lipid peroxidation (MDA) colorimetric assay kit (Abcam, Prodotti Gianni S.p.A., Milan, Italy). Each sample was assayed in duplicate, following manufacturer’s instructions and using a Multiskan Go multiwell plate reader (Thermo Fisher Scientific, Waltham, MA, USA).

### 2.11. NQO1 and CYP3A Enzymatic Activity

Furthermore, we investigated the catalytic activity of two key enzymes involved in the antioxidant response and in AFB1 bioactivation, i.e., NQO1 (NAD(H):quinone oxidoreductase 1) and CYP3A, here reported to be transcriptionally modulated by treatments.

NQO1 activity was measured using the ab184867 NQO1 activity assay kit (Abcam), using the same protein extract isolated for the MDA assay (see above).

CYP3A catalytic activity was measured using the P450-Glo^TM^ CYP3A4 assay with Luciferin-IPA (Promega Corporation, Madison, WI, USA) and following the manufacturer’s protocol. BFH12 cells were seeded in opaque white P96 multi-well plates (Corning, New York, NY, USA; catalogue 3917) at a density of 6 × 10^3^ cells/well and treated as reported in Figure 1. Six independent cell culture experiments were performed in sextuplicate. At the end of the incubation time, the medium was removed, cells were washed with PBS buffer and incubated with luciferin-IPA substrate at 37 °C in serum-free medium for 1 h. The reaction was terminated by transferring 25 μL of the final incubation mixture from each well to a separate plate with wells containing 25 μL of luciferin detection reagent. After 20 min at room temperature, the luminescence signal was measured by VICTOR™X4 Multilabel Plate Reader (Perkin Elmer, Waltham, MA, USA). Cells were then assayed for cell number using ATP as a marker for viability (CellTiter-GloTM cell viability assay kit, Promega). Luminescence (RLU) was finally expressed as normalized to cell number.

### 2.12. Statistical Analysis

Dose–response curves were obtained by using GraphPad Prism software (version 8.0.2, San Diego, CA, USA). Mortality rates of the treated cells compared to cells exposed to the vehicle only (DMSO) were reported, and a nonlinear regression [log(inhibitor) vs. normalized response, variable slope] was built. The IC_50_ and the goodness of fit (R squared) were provided by the software.

Analytical data were analyzed with GraphPad Prism using an ordinary one-way ANOVA followed by Tukey’s multicomparisons test with the level of significance set at *p* ≤ 0.05.

The statistical analysis of qPCR data, MDA content, and enzymatic activities (i.e., NQO1 and CYP3A) were performed by using the one-way ANOVA followed by Dunnett’s multicomparisons test with the level of significance set at *p* ≤ 0.05.

## 3. Results

### 3.1. Curcuminoids Cytotoxicity

**Figure 2:**
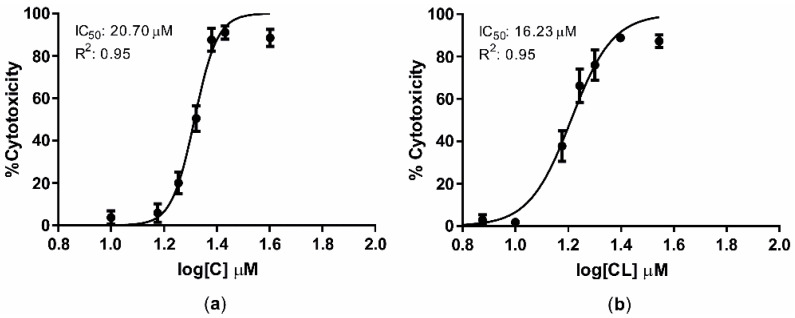
Dose–response curves of C (**a**) and CL (**b**) after the incubation of BFH12 cells for a total of 64 (16 + 48) h. Graphs were obtained by means of GraphPad prism software and using three independent cell culture experiments, each one run in sextuplicates. Data are expressed in mean cytotoxicity rate ± mean standard error (SEM). IC_50_ and R^2^ are also reported. C = curcumin; CL = *Curcuma longa*.

Cells were exposed for 16 + 48 h to increasing C and CL concentrations and dose/response curves were built to define the corresponding IC_50_ values. After 64 h of incubation, IC_50_ values were 20.70 μM (R^2^ = 0.95) and 16.23 μM (R^2^ = 0.95) for C and CL, respectively (Figure 2).

**Figure 3:**
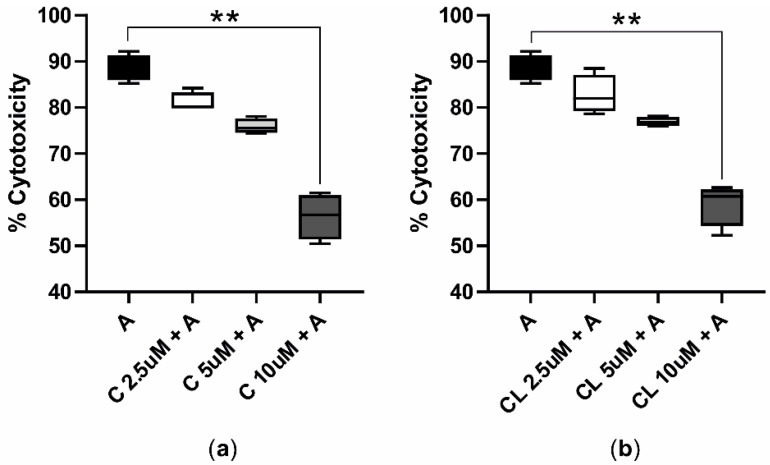
AFB1 cytotoxicity in presence of C or CL (16 + 48 h). The box and whiskers plots report the viability of BFH12 cells pretreated with C (**a**) and CL (**b**) increasing concentrations (2.5, 5, and 10 μM) and exposed for 48 h to a combination of AFB1 3.6 μM and C or CL (same concentrations reported above). ** *p* ≤ 0.01 (Kruskal–Wallis and Dunn’s multicomparisons tests; the mean of each condition was compared with the mean of the AFB1 condition). C = curcumin; CL = *Curcuma longa*; A = AFB1.

The cytotoxicity assessed following the AFB1 cotreatment with C or CL showed that curcuminoids mitigated AFB1-induced cytotoxicity in a dose-dependent manner. In particular, the treatment with the highest dose of C and CL (i.e., 10 μM) significantly reduced the median AFB1 cytotoxicity, dropping from 88.37% to 56.70% (Figure 3a) and 60.71% (Figure 3b), respectively.

### 3.2. Aflatoxin B1 Biotransformation in BFH12 Cells

Bovine fetal hepatocytes were proved to be able to metabolize AFB1, producing and releasing into the medium its foremost derivatives, i.e., AFM1 and AFL. The amount of AFL detected into the medium was the same in all the experimental conditions and close to 100 ng/mL. On the contrary, curcuminoids hampered the production of AFM1 in a dose-dependent manner (Figure S1). Aflatoxin M1 and AFL were not detected in cellular pellets, while the amount of cellular AFB1 was very low (approximately 1.5 ng/mL) and not statistically different between the experimental conditions.

### 3.3. Target Gene Expression Analysis

Gene expression data revealed that some key genes involved in phase I and II biotransformation, AHR pathways, and antioxidant mechanisms were affected by curcuminoids, administered alone and/or in cotreatment with AFB1 (Figures S2 and S3). Overall, all target gene mRNA levels except for CYP1B1, ARNT, and KEAP1, were modulated by C and CL. Specifically, curcuminoids upregulated CYP1A1, NQO1, AHR, and SOD1. On the contrary, mRNA levels of CYP3A28 (the orthologue of human CYP3A4, as demonstrated in [54]), NRF2, CAT, GPX1, and SOD2 were negatively affected by C and/or CL. The observed expression patterns demonstrated that the curcuminoids effects are dose-dependent, and the highest dose (i.e., 10 μM) produced maximal variations. In order to obtain net transcriptional results, the RNA-seq studies were carried out only in cells treated with the highest C and CL concentrations.

### 3.4. Whole-Transcriptome Differential Expression Analysis

A total of 510,467,752 raw reads were obtained and deposited in GeneBank under the BioProject accession PRJNA627332.

All samples passed quality control measures for raw sequenced reads. After trimming and rRNAs removal, an average of about 28 million reads per sample were retained, with ≈99% of reads mapping to the *B. taurus* reference genome. Number of raw reads passing the filters, and number of filtered reads mapping to the cow genome are provided in Table S2. The MDS plot reported in Figure S4 provides an unsupervised clustering of the samples. The first dimension (x axes) clearly separates AFB1-treated cells from control cells, being cells exposed to curcuminoids alone or in combination with AFB1 in an intermediate position. Biological variability within CTRL, AFB1, C, and CL experimental groups is very low as demonstrated by the coherent clusters formed by replicas. Notably, cells treated with C or CL have a very similar transcriptional profile, thus forming a unique cluster in the plot. On the contrary, samples cotreated with curcuminoids and AFB1 are more dispersed in the Cartesian plane, thus suggesting a certain biological variability between replicas.

#### 3.4.1. Transcriptional Effects of Curcuminoids

In cells exposed to C, a total of 920 differentially expressed genes (DEGs) were detected; compared to controls, 529 and 391 were up- and downregulated, respectively. Likewise, the treatment with CL induced the expression of 312 genes and inhibited the transcription of 152 genes, for a total of 464 DEGs. The EdgeR output of the DE analysis conducted in this study was reported in Table S3.

**Figure 4:**
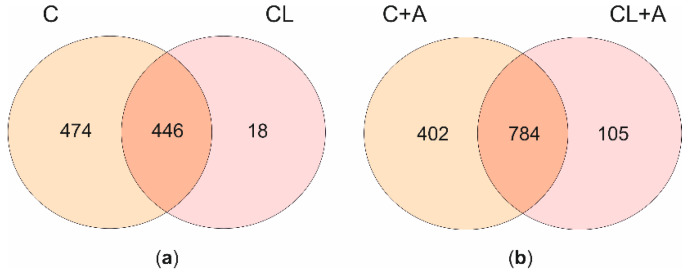
Transcriptional effects of curcuminoids. The Venn diagrams report the number of unique and shared DEGs after C and CL treatments (**a**), and after C + A and CL + A cotreatments (**b**). C = curcumin; CL = *Curcuma longa*; A = AFB1.

When comparing the two lists of DEGs, a core of 446 genes appeared to be significantly regulated by both treatments with minor differences in terms of FC; however, 474 were exclusively affected by C, and 18 by CL (Figure 4a). The core of 446 genes showed the same transcriptional pattern (i.e., up- or downregulation) after the exposure to both curcuminoids. Overall, the whole transcriptional profiles of cells exposed to either C or CL were extremely similar, as demonstrated by the correlation analysis carried out using as input the log_2_FC of all the expressed genes resulting from the edgeR analysis comparing C vs. CTRL and CL vs. CTRL (Figure S5a). Moreover, nine out of the top-10 (90%) up- and downregulated genes by C, ranked according to FDR, were also listed among the top-10 up- and downregulated genes by CL (Table S4).

**Figure 5:**
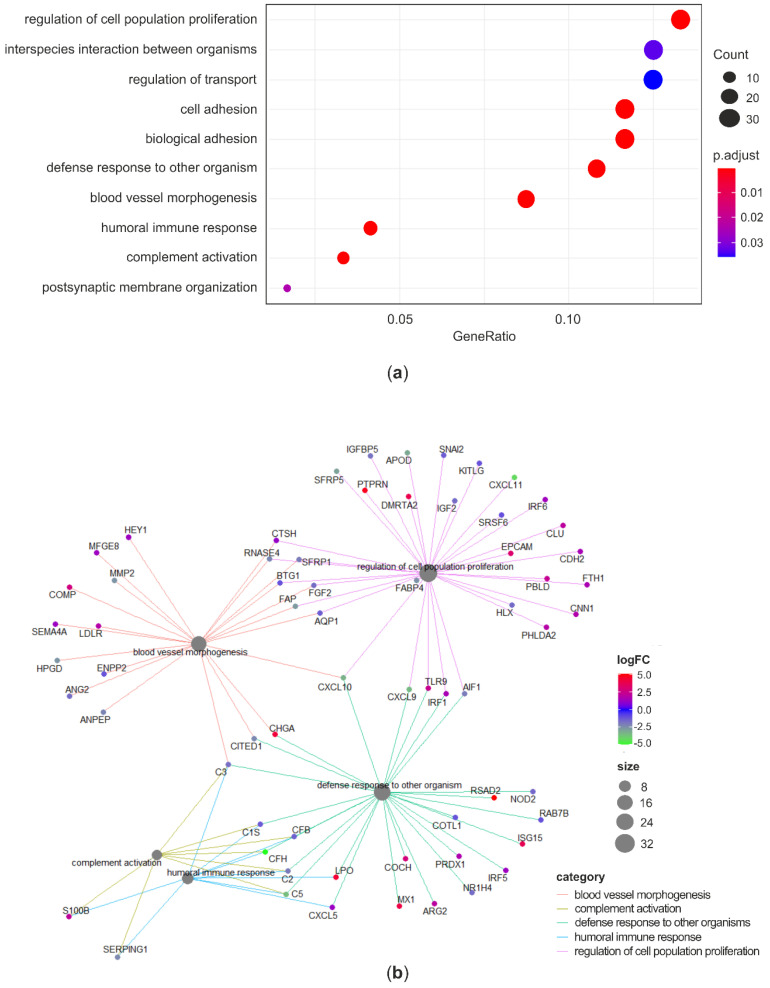
Gene ontology analysis: C vs. CTRL. Dotplot reporting the over-represented BP terms in the list of DEGs between C and CTRL (**a**). Gene-concept network linking DEGs and enriched BP terms (**b**). GO terms redundancy was removed by using the *simplify* method (similarity cutoff = 0.5). ‘Count’ is the number of genes enriched in a GO term. ‘Gene ratio’ is the percentage of total DEGs in the given GO term. *p*-values were adjusted using the Benjamini–Hochberg method.

As C significantly changed the expression of a vaster number of genes compared to CL, and the ability of mitigating AFB1-induced cytotoxicity in BFH12 was higher for C compared to CL, only the GO enrichment carried out on the list of DEGs (both up- and downregulated) resulting from the comparison between C and CTRL was discussed in detail. The GO over-representation test performed in the list of 920 significant DEGs found a total of 40 enriched terms (Table S5). The enriched biological process (BP) terms were represented in a dotplot reporting gene ratios and adjusted *p*-values (Figure 5a). The analysis highlighted an over-representation of terms related to cell proliferation, cell adhesion, and immune system regulation. The gene-concept network reported in Figure 5b pointed out some relevant genes involved in the top significant BP terms. Notably, among the key genes implied in the immune and defense response processes and whose expression was inhibited by C there are the nucleotide-binding oligomerization domain-containing protein 2 (NOD2), the allograft inflammatory factor 1 (AIF1), some complement components (C1S, CFB, CHF, C2, C3, C5), the cell motility promoting factor ENPP2, and the transcriptional and immune response regulator (TCIM). On the contrary, some genes related to immunity were upregulated following C treatment. For instance, the expression of antiviral genes like viperin (RSAD2), the myxoma resistance protein 1 (MX1), the interferon alpha-inducible protein (ISG15), the interferon regulatory factor 5 (IRF5) were induced to a greater extent. Likewise, genes involved in arginine metabolism (ASS1 and ARG2) and antioxidant response like lactoperoxidase (LPO), peroxiredoxin 1 (PRDX1), apoferritin (FTH1), and heme oxigenase 1 (HMOX1) were upregulated in C-treated cells. Regarding key detoxification enzymes, mRNA levels of CYP2B6 as well as of some GSTs (i.e., GSTA1, GSTM1, GSTM3) were significantly increased by C treatment. Finally, genes involved in cells adhesion and proliferation, such as versican (VCAN), insulin like growth factor 2 (IGF2), and KIT ligand (KITLG) were found to be significantly downregulated.

Interestingly, among the top-10 upregulated genes, we found calpain 5 (CAPN5) and cystatin B (CSTB), or stefin B. These genes are associated to cell death, together with cathepsins (CTSs). Notably, some CTSs, i.e., CTSH, CTSC, CTSL, and CTSZ, were upregulated, while CTSF was downregulated by C.

For the sake of completeness, the results of the GO over-representation test performed on the list of DEGs obtained from the comparison between CL and CTRL was reported in Table S5.

#### 3.4.2. Effects of Curcuminoids on AFB1-Induced Transcriptional Changes

Transcriptional profiles of cells treated with AFB1 were compared to those of cells cotreated with curcuminoids and AFB1. In cells cotreated with C and AFB1, a total of 1186 DEGs were identified, of which 809 were upregulated and 377 downregulated. Similarly, the treatment with CL induced the expression of 656 genes and inhibited the transcription of 233 genes. Overall, a total of 889 DEGs were found in cells exposed to CL. The EdgeR output of the DE analysis conducted in this study is reported in Table S3. The two lists of DEGs were compared. A core of 784 genes appeared to be significantly regulated by both treatments, 402 were exclusively affected by C, and 105 responded solely to CL (Figure 4b). The core of 784 genes showed the same transcriptional pattern after C or CL treatment. As reported for cells exposed to curcuminoids only, the overall transcriptional profiles (i.e., all the expressed genes) of cells cotreated with AFB1 and C or CL were extremely similar (Figure S5b).

**Figure 6:**
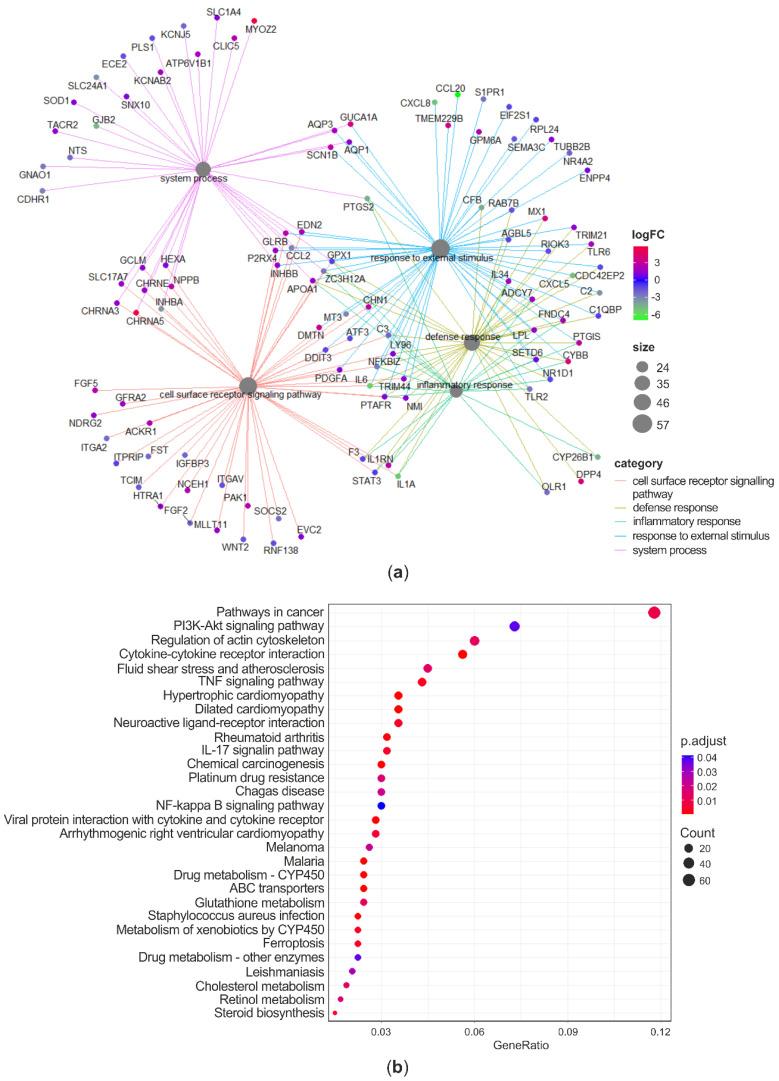
Gene ontology analysis: C + AFB1 vs. AFB1. Gene-concept network linking DEGs and enriched BP terms (**a**). Dotplot reporting the over-represented KEGG pathways in the list of DEGs between C + AFB1 and AFB1 (**b**). ‘Count’ is the number of genes enriched in a KEGG pathway. ‘Gene ratio’ is the percentage of total DEGs in the given pathway. *p*-values were adjusted using the Benjamini–Hochberg method.

Following the approach adopted to investigate the effects of curcuminoids alone, the gene ontology analysis was carried out only on the list of DEGs (both up- and downregulated) between C + AFB1 and AFB1 conditions. The GO over-representation test, performed in the list of 1186 significant DEGs, found a total of 30 enriched terms (Table S5). Several BP terms related to immunity, inflammation, and defense response were found to be significantly over-represented (e.g., “inflammatory response”, “response to external stimulus”, “defense response”, “immune system process”, “response to cytokine”). The top-5 enriched BP and their related DEGs were reported in a gene-concept network (Figure 6a). As far as the inflammatory and defense response are concerned, complement components (CFB, C2, C3), interleukins 1A and 6 (IL1A, 1L6), prostaglandin-endoperoxide synthase 2 (PTGS2 or COX2), nuclear factor kappa B subunits 1 and 2 (NFKB1, NFKB2) were downregulated. On the other hand, fibronectin type III domain-containing protein 4 (FNDC4), IL34, and myeloid differentiation protein-2 (LY96), were upregulated. With regards to the enriched BPs associated to response to stimulus (“cellular response to chemical stimulus” and “response to external stimulus”) the expression of phospholipase A2 receptor 1 (PLA2R1) and phospholipase A2 group IVA (PLA2G4A), the Fas cell surface death receptor (FAS), the chemokine (C-X-C Motif) ligands 5 and 8 (CXCL5, CXCL8), and SOD2 were inhibited in C + AFB1 condition. These significant enriched BPs included also the colony stimulating factor 3 (CSF3), which was listed among the top-3 downregulated genes, with a considerable log_2_FC equal to −8.19. Some examples of significantly upregulated genes were thioredoxin reductase 1 (TXNRD1), SOD1, P21 (RAC1) activated kinase 1 (PAK1), and integrin subunit beta 2 (ITGB2).

Looking at the 30 over-represented KEGG pathways (Figure 6b, Table S6), it is confirmed that several DEGs between C + AFB1 and AFB1 conditions are involved in carcinogenesis (i.e., “pathways in cancer”, “chemical carcinogenesis”), immunity and inflammation (e.g., “cytokine-cytokine receptor interaction”, “TNF signaling pathways”), and drug metabolism (e.g., “drug metabolism–cytochrome P 450”, “ABC transporters”). A further interesting enriched pathway was “ferroptosis”, suggesting that C affected genes playing a role in this cell death event usually accompanied by lipid peroxidation, a marker of oxidative stress. The presence of some pathways related to pathogens infection, i.e., “malaria”, “Chagas disease”, “*Staphylococcus aureus* infections”, and “Leishmaniasis” refers more in general to genes involved in the immune response (e.g., complement activation, interleukins). Likewise, the pathway “Rheumatoid arthritis” is associated to inflammatory genes (e.g., chemokine (C-X-C motif) ligands, interleukins).

The output of the KEGG GSEA conducted on the complete list of expressed genes was consistent with the results of the over-representation analysis performed on the DEGs only (Figure S6, Table S7). Indeed, KEGG pathways like “drug metabolism–cytochrome P450”, “metabolism of xenobiotics by cytochrome P450”, and “lysosome” were significantly activated in C + AFB1 compared to AFB1 condition. Several terms related to immunity appeared to be significantly suppressed by C treatment. Some examples are “IL-17 signaling pathway”, “TNF signaling pathway”, and “NF-kappa B signaling pathway”. As far as carcinogenesis is concerned, “p53 signaling pathway” and “bladder cancer” were listed among the suppressed KEGG terms.

Finally, if comparing genes significantly regulated after C treatment with those regulated after C and AFB1 cotreatment, out of 1186 DEGs, a core of 248 genes (i.e., 21%) was found.

### 3.5. Oxidative Stress

**Figure 7:**
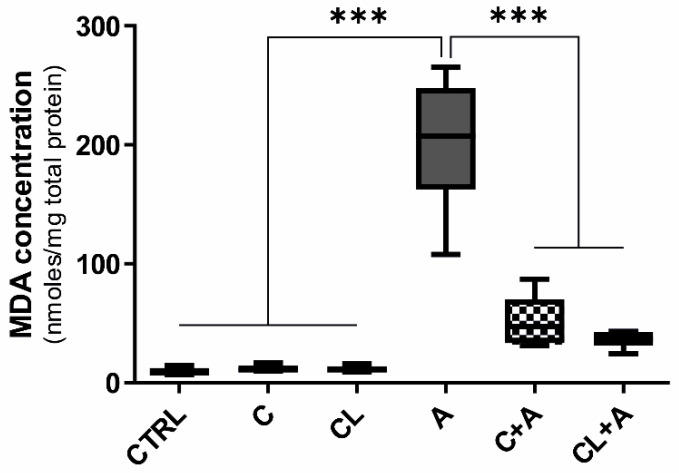
Lipid peroxidation (MDA content). Box and whiskers plot reporting the MDA content in the different experimental conditions. The median MDA concentration in cells exposed to AFB1 was compared to the MDA concentration observed in all the other experimental conditions. Data are expressed as median concentration and data distribution (i.e., quartiles) of six independent cell culture experiments. CTRL condition corresponds to cells exposed to PCB126 only. C = curcumin; CL = *Curcuma longa*; A = AFB1. *** *p* < 0.001 (one-way ANOVA, followed by Dunnett’s multicomparisons test).

Lipid peroxidation is one of the main manifestations of oxidative damage; moreover, it is known to play an important role in AFB1 toxicity; therefore, we measured the amount of MDA, a product of lipid peroxidation, in all the experimental conditions. A significant increase of MDA was observed in BFH12 cells exposed to AFB1 compared to control conditions (Figure 7). Interestingly, the coincubation with both C and CL significantly reduced the MDA content (Figure 7), thus demonstrating the potential of curcumin in mitigating AFB1-induced oxidative damage.

### 3.6. NQO1 Enzymatic Activity

Diaphorase or NQO1 is a multifunctional and stress-inducible dimeric protein involved in the antioxidant defense. It is a FAD-binding protein that is employed in the removal of a quinone from biological systems as a detoxification reaction, reducing quinone to hydroquinone, thus avoiding the formation of semiquinones and species with reactive oxygen radicals that are deleterious to cells [55]. In this study, the activity of this enzyme was selected as a marker of the cell antioxidant status and, additionally, to confirm the results obtained for this specific target at the mRNA level (qPCR and RNA-seq: Figures S2 and S3, Table S3).

**Figure 8:**
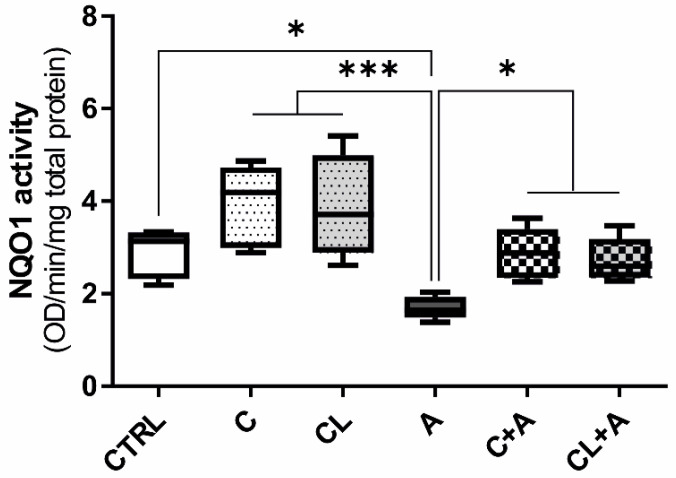
NQO1 enzyme activity. Box and whiskers plot reporting the NQO1 activity in the different experimental conditions. The median NQO1 activity in cells exposed to AFB1 was compared to the activity observed in all the other experimental conditions. Data are obtained from six independent cell culture experiments and expressed as median optical density (OD) observed per min per mg of total protein. CTRL condition corresponds to cells exposed to PCB126 only. C = curcumin; CL = *Curcuma longa*; A = AFB1. * *p ≤* 0.05; *** *p ≤* 0.001 (one-way ANOVA, followed by Dunnett’s multicomparisons test).

The treatment with AFB1 significantly reduced the antioxidant activity of NQO1, compared to CTRL condition (Figure 8). However, the use of both C and CL, in combination with AFB1, significantly induced the NQO1 enzymatic activity, bringing it back to CTRL levels (Figure 8). This result further corroborates the antioxidant properties of curcumin.

### 3.7. CYP3A Enzymatic Activity

The cytochrome P450 3A plays a pivotal role in the bioactivation of AFB1 and in the generation of the highly reactive epoxide intermediate AFB(1)-8,9-epoxide [11]. Having noticed in the preliminary part of this study a modulation of CYP3A28 mRNA expression using both qPCR and RNA-seq approaches (Figures S2 and S3, Table S3), we measured CYP3A activity in all the experimental conditions as a confirmatory step.

**Figure 9:**
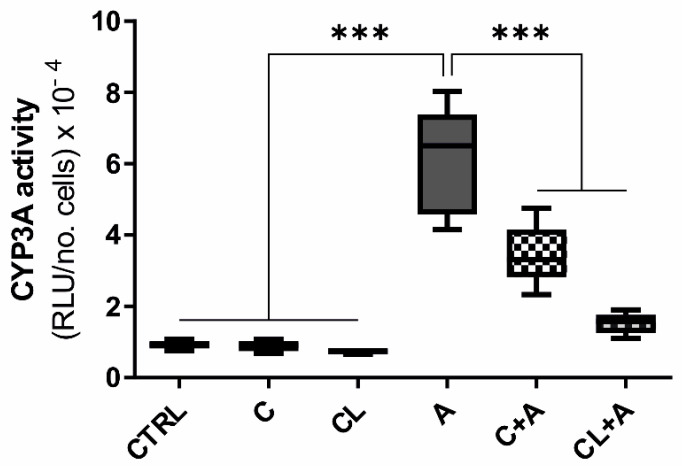
CYP3A enzyme activity. Box and whiskers plot reporting the CYP3A activity in the different experimental conditions. The median CYP3A activity in cells exposed to AFB1 was compared to the activity observed in all the other experimental conditions. Data are obtained from six independent cell culture experiments and expressed as relative luminescence units (RLU) normalized to the total number of alive cells. CTRL condition corresponds to cells exposed to PCB126 only. C = curcumin; CL = *Curcuma longa*; A = AFB1. *** *p* ≤ 0.001 (one-way ANOVA, followed by Dunnett’s multicomparisons test).

Cells exposed to AFB1 alone showed a statistically significant increase in CYP3A activity compared to basal conditions (*p ≤* 0.001, Figure 9). Conversely, the combined exposure to AFB1 and C or CL considerably reduced the CYP3A activity, confirming the same effect observed at the gene expression level.

## 4. Discussion

The molecular mechanisms triggered by curcuminoids and underlying their protective activity against aflatoxicosis have never been investigated in depth. Whole-transcriptomic changes resulting from the cotreatment of human hepatocytes with curcuminoids and AFs are still unknown. Regarding susceptible livestock species, some studies assessing in vivo the protective effects of curcumin against AFB1 in poultry have been published [33,36,39,40,41,42]. However, none of them employed a whole-transcriptomic approach. To the best of our knowledge, this is the first study evaluating the whole mRNA profiles of bovine liver cells coexposed to curcuminoids and AFB1, and providing important information to tackle aflatoxicosis in cattle.

Since curcumin is typically administered in the form of turmeric powder, we assessed both the high purity compound (C) and the extract from *C. longa* (CL), that also contains the curcumin main derivatives bisdemethoxycurcumin and demethoxycurcumin. Interestingly, C showed greater effects in terms of both cell viability (and transcriptional regulation as well), most likely due to the different percentage of curcumin (i.e., ≥94% and ≥65% in C and CL, respectively). Notably, this is in accordance with the fact that curcumin possesses a higher antioxidant capacity compared to its metabolites [56].

### 4.1. Curcuminoids Cytotoxicity

Half maximal inhibitory concentration (IC_50_) at 64 h was roughly similar between the two compounds, and in partial agreement with the results obtained in human cell lines [49,57,58]. Conversely, contradictory results were reported in human liver cancer HepG2 cells, in which curcumin exhibited an IC_50_ of 23.15 μM and 8.84 μM after 24 h of treatment [59,60]. Interestingly, bovine mammary epithelial cells (BME-UV1) proved to be more sensitive compared to BFH12 cells, as C and CL half maximal lethal concentration (LC_50_), after 48 h of incubation, was in the range of 10–20 and 5–10 μM, respectively [20]. Overall, curcumin IC_50_ has been reported to be extremely variable depending on the cell type and the time of treatment, ranging from 2 to 40 μM [61].

Both C and CL significantly decreased the AFB1-induced cytotoxicity in a dose-dependent manner. Notably, in BME-UV1 cells, only 5 μM CL provided some protection against AFB1-induced cytotoxicity, increasing cell viability up to 40%, while 5 μM C did not affect cell viability at any experimental conditions [20]. Such evidence let us hypothesize that the potential benefits of curcumin in cattle might depend on the target tissue and the relative abundances of AFB1 as well as of curcumin molecular targets.

### 4.2. Aflatoxin B1 Biotransformation

Although the characterization of AFB1 biotransformation was not the primary goal of our study, some results are worth noting. As discussed in a previous study [43], a small amount of AFB1 was metabolized by BFH12 to produce and release into the medium AFM1 and AFL. In particular, the amount of AFM1 appeared inversely correlated to C and CL concentrations. This is of particular interest since aflatoxin M1 is the most toxic AFB1 derivative [62], classified in Group 2B carcinogens [63]. The molecular mechanisms by which C and CL can reduce AFM1 production most probably target CYP1A and/or CYP3A expression, translation and/or activity. Indeed, in bovine hepatocytes these enzymes are known to be involved in AFM1 formation [64]. Taking into consideration CYP1A and 3A gene expression results and the observed CYP3A enzymatic activity, it is likely that CYP3A28 is the foremost CYP involved in AFM1 synthesis in BFH12 cells. In fact, the AFM1 decrease occurring in presence of C and CL is consistent with the observed reduced transcription and catalytic activity of CYP3A as well as with the increase of CYP1A1 mRNA (compared with cells exposed to AFB1 only). We might hypothesize curcuminoids target CYP3A28 by reducing its transcription and/or competing with AFB1 for binding to the catalytic site, ultimately resulting in minor AFM1 formation. Nonetheless, it has been previously demonstrated in rat [65] and human [66] liver, as well as in human intestinal cells [67], that curcuminoids have the potential of reducing CYP3A activity.

The aflatoxin B1 absorption by BFH12 cells was demonstrated by the amount of AFB1 detected in cellular pellets, although it was very low and close to 1.5 ng/mL.

### 4.3. RNA-Seq Analysis of Curcuminoids-Exposed Cells

Comparing C and CL, greater variations in BFH12 transcriptome (i.e., twice as many DEGs) were observed in cells exposed to C. Hence, curcumin is most likely mainly responsible for the observed transcriptional effects. Looking at the DEGs affected by both curcuminoids, several lines of evidence suggest that C and CL caused very similar effects, differing only in their magnitude. If compared with pure curcumin, turmeric (i.e., *C. longa*) does not require complex and expensive extraction and concentration procedures. This makes CL particularly interesting as a feed additive. However, assuming the differences between the two treatments essentially due to a different curcumin concentration, and considering the goal of the present study (mechanistic-oriented), only the effects resulting from C exposure will be hereby discussed more in depth.

Regarding DEGs associated with immune system, several complement components were inhibited by C. Complement components are among the most important proinflammatory molecules and a decrease in their transcription reflects an anti-inflammatory activity mediated by C, already suggested in previously published studies (e.g., [68,69]). The possible C-dependent anti-inflammatory activity is someway corroborated by additional evidence, like the downregulation of proinflammatory genes. Some examples are the TNF Alpha Induced Protein 6 (TNFAIP6) and NOD2, known to be regulated by C [70] as well as IGF2. This latter gene has been previously demonstrated to be a key player in C-mediated inhibition of urothelial tumor development [71].

Several DEGs functionally included in the immune system/response terms are involved in antioxidant functions, the hallmarks of C. Genes coding for enzymes with antioxidant properties, such as LPO, PRDX1, TXN, glutaredoxin (GLRX), FTH1, and HMOX1 were significantly upregulated. Notably, scientific literature has largely demonstrated that C cytoprotective effects are also mediated by the upregulation of HMOX1 [72]. Indeed, in monocyte and macrophage cellular models, a C-mediated upregulation of HMOX1 contributes to inhibit inflammation (e.g., [73,74]) and blocks apoptosis in cardiac myoblasts [75]. In rat primary hepatocytes, the pretreatment with 0–50 μM C alleviates ethanol-induced oxidative damage through a HMOX1 induction [76].

The balance between pro- and antiapoptotic processes appears as a fundamental issue in BFH12 cells treated with C. Two DEGs listed in the top-5 upregulated genes, namely CAPN5 and CSTB, belong to calpains and cathepsins families of proteins, known to play a role in lysosomal membrane permeabilization and activation of caspase-8, secondary events of apoptosis induction. This mechanism has been hypothesized in human hepatoma-derived Huh-7 cells exposed to C, and it supports the anticancer potential of this polyphenolic compound [77]. Indeed, apoptosis might represent a fail-secure mechanism to prevent cancerous cells proliferation. Cystatin B acts in the opposite direction, as it is the major endogenous intracellular inhibitor of cathepsins, and most probably represents a mechanism to counterbalance an extensive programmed cell death.

Regarding the major antioxidant enzymes, transcriptional profiles of GPX, SOD, and CAT were not affected. Although GPX was not modulated, two genes coding for enzymes playing a role in GSH synthesis, i.e., glutathione-disulfide reductase (GSR) and glutamate-cysteine ligase catalytic subunit (GCLC), were significantly induced by C. This evidence lets us think that GSH synthesis may be involved in the mechanism of action of C, as demonstrated in a recent study assessing the effects of this polyphenol in tilapia hepatocytes [78].

An additional interesting result is the upregulation of GSTs, and particularly GSTA1. Such an induction has been previously demonstrated and postulated to be a helpful mechanism for colon cancer chemoprevention [79]. In BME-UV1, 5 μM C alone did not affect GSTA1 and GSTA2 mRNA levels, evaluated by using a qPCR approach [20], thus indirectly suggesting an organ-specific activation of these phase II detoxification enzymes in cattle.

For a comprehensive understanding of the expression pattern of all the above discussed genes, the mean CPM of each experimental group has been added in Table S8.

### 4.4. RNA-Seq Analysis of Cells Coexposed to C and AFB1

Curcumin deeply modified the whole transcriptome of cells treated with AFB1, and these variations reflected the activation of molecular mechanisms conferring protection against AFB1 toxicity. In particular, the regulation of genes and biological processes related to antioxidant response, defense response, inflammation, carcinogenesis, and drug metabolism appeared pivotal to cope with AFB1-induced cell toxicity and death. These genes and biological processes are hereby more specifically discussed, while their gene expression level in all treatments is reported in Table S8.

#### 4.4.1. Antioxidant Response

Aflatoxin B1 causes oxidative stress and eventually the generation of free radicals and cellular damage [80]. These harmful effects can be counteracted by effective antioxidant enzymes offering protection against cellular damage by scavenging radical compounds. In the present study, some antioxidant mechanisms were triggered by C, thus conferring protection against AFB1 toxicity. Notably, AFB1-induced oxidative damage was here confirmed by an increased lipid peroxidation, measured as MDA content. Indeed, the potential of curcuminoids treatment (C and CL) in reducing AFB1-dependent oxidative damage was confirmed by their ability of dropping MDA content approximately to CTRL levels, as previously reported in chick [37,40] and rat liver [30].

Superoxide dismutase 1 and 2 showed an opposite transcriptional behavior. Superoxide dismutase 1 is the foremost cellular SOD; moreover, it is widely recognized as a target of C. In chicken fibroblasts, 10–40 μM C reversed the SOD1 downregulation due to heat stress [81]. Rats exposed to diethyl nitrosamine exhibited a decrease in hepatic SOD1 mRNA levels but C, daily administrated via intra-gastric intubation (100–300 mg kg^−1^ body weight for 20 days), significantly brought SOD1 mRNA amounts back to physiological levels [82]. On the contrary, in BME-UV1 cells, the coincubation of 375 nM AFB1 with 5 μM C did not result in appreciable changes in SOD1 gene expression [20]. We hypothesize this latter finding is likely due to the lower concentrations of both AFB1 and C compared to our study and/or differences in sensitivity of the two cattle cell lines.

As regards SOD2, our results are in contrast with most of the literature that reports this enzyme similar behavior to SOD1 (e.g., [81,83]) and thus concurring in radical species scavenging. It should be noted that C itself has a potential toxic effect on cells and, though the concentration employed in this study is very low, it is still possible that some pro-oxidant effects appear. Nonetheless, here we focused on transcriptional modifications, but post transcriptional regulations might occur (i.e., protein expression, catalytic activity) and affect the overall antioxidant capacity of cells.

As observed for SOD2, GPX1 was inhibited by C treatment. This enzyme, together with SODs, counteracts oxidative stress, and the present transcriptional result is in opposition to most of published studies assessing C transcriptional effects. Nonetheless, curcumin might also be responsible for a GPX1 downregulation, as demonstrated in the liver of hypothyroid rats fed C for 30 days (30 mg/kg body weight) [83].

Looking at NRF2, a master regulator of the antioxidant response controlling the cytoprotective defense system [84], its expression was not significantly changed after C treatment. A similar behavior was noticed in another bovine mammary epithelial cell line (MAC-T), in which resveratrol reversed AFB1 toxicity without affecting NRF2 transcription [85]. Despite this unexpected result, the transcription of some genes downstream regulated by NRF2 were significantly impacted by C, as previously reported in literature (e.g., [86,87]). Among them, NQO1 (or diaphorase) is one of the most important actors. In a former study in which BFH12 cells were exposed only to AFB1, the NQO1 gene was downregulated [43], and a similar behavior was also noticed in the present study; interestingly, the C treatment counteracted NQO1 negative modulation at both mRNA level and protein activity. Diaphorase catalyzes a two-electron reduction of many environmental electrophilic contaminants and endogenous compounds, thus preventing the generation of ROS (reactive oxygen species) and scavenging oxygen radicals. Therefore, NQO1 plays a direct role in protection against oxidative stress [88]. Notably, previous studies have demonstrated this gene is inhibited by several stressors, but the treatment with some natural antioxidants is able to counterbalance this downregulation. For instance, a long-term administration of resveratrol (0.3 and 0.6 g/kg diet for 60 days) significantly reactivated NQO1 transcription in tilapia suffering of an oxidative stress-induced liver damage [89]. Interestingly, in MAC-T cells treated with 12.81 μM AFB1 and 43.81 μM resveratrol, NQO1 mRNA levels were significantly higher when compared to those measured in cells treated with AFB1 alone [85]. Worth mentioning, the modulatory effects of C on NQO1 have been previously described also at the post-transcriptional level (protein expression [90,91,92] and catalytic activity [93]).

Another NRF2 downstream gene upregulated by C treatment is HMOX1, an antioxidant gene playing a protective role in several pathologies (e.g., [86,94,95]), and reported to be a molecular target of C [96]. Together with HMOX1 upregulation, we observed an increasing expression of two additional NRF2-dependent genes, encoding for components of the ferritin complex: the ferritin light and heavy polypeptides (FTL and FTH1, respectively). This is a well-known mechanism to prevent hydroxyl radical formation resulting from HMOX1 activation (e.g., [97]). Notably, it has also been postulated that an activation of HMOX1 might result in an indirect minor NFKB activation [98,99]; this hypothesis substantially agrees with the concomitant NFKB1 and NFKB2 downregulation observed in the present study and leading to anti-inflammatory and antiapoptotic effects.

Regarding the GSH and TXN antioxidant system, some key genes transcriptionally controlled by the NRF2 pathway were upregulated by C treatment, i.e., the glutamate-cysteine ligase (GCLM), several GSTs (GSTA1, A2, M1, and M3), TXNRD1, and sulfiredoxin 1 (SRXN1). The former gene (GCLM) codes for an enzyme catalyzing the rate-limiting step of GSH synthesis, while GSTs are in charge of ROS detoxification. Both TXNRD1 and SRXN1 are essential for the reduction of protein thiols [100].

Finally, C treatment increased, via a NRF2 activation, the expression of two important NADPH-regenerating enzymes: the isocitrate dehydrogenase 1 (IDH1) and the malic enzyme 1 (ME1). These enzymes are of crucial importance because NADPH is an obligatory cofactor for many drug-metabolizing (e.g., CYPs) and antioxidant (e.g., NQO1) enzymes [101].

#### 4.4.2. Defense Response and Inflammation

The “condition of oxidative stress”, and thus the resulting cell damage, is closely associated to the inflammatory response [102]. In our experimental conditions, the obtained RNA-seq data showed that C has the potential to counteract some transcriptional changes triggered by AFB1 and attributable to a proinflammatory reaction. Some genes significantly regulated by C treatment and implied in inflammation have been discussed above.

Interleukin 1A and IL6 mRNA levels were significantly downregulated when BFH12 cells exposed to AFB1 were treated with C, thus mitigating the AFB1-induced inflammatory response. Notably, an AFB1-induced toxic proinflammatory response, mediated by the increase of IL1A and IL6, has been previously noticed in macrophages collected from rats orally consecutively exposed to AFB1 for 2 weeks (corresponding to 0.03–0.7 mg/kg body weight) [103], in murine CNS-derived cells treated with their relative AFB1 LC_50_ dose [104], as well as in a rat model of HCC intraperitoneally injected with 1 mg/kg AFB1 [105].

Confirming what was mentioned above, NFKB1 and NFKB2, two genes playing a pivotal role in mediating the inflammatory response and shown to be induced by AFB1 [43], were inhibited by C treatment. Overall, the inhibition of the transcription factor NFKB mediated by C has been previously reported in several cellular models [106,107] and, together with the activation of NRF2 pathway, represents one of the key mechanisms by which C protects cells from liver injury [86]. Interestingly, a natural compound endowed with beneficial properties, i.e., the grape seed proanthocyanidin, showed protective effects against the AFB1-induced immune system activation by suppressing the inflammatory response and inhibiting the NFKB expression in broilers spleen [108]. Furthermore, the transcription of PLA2G4A and COX2, two key enzymes mediating prostaglandin synthesis in inflammatory cells, and known to be upregulated by AFB1 [43], were significantly decreased in C + AFB1 condition. In inflammation, the arachidonyl specific group IVA cytosolic PLA2 enzyme (cPLA2α), encoded by the PLA2G4A gene, is a major contributor to the increased levels of arachidonic acid (AA), a precursor of cyclooxygenase-mediated prostaglandins synthesis. In particular, COX2 has been shown to play a pivotal role in inflammation and carcinogenesis, and its inhibition represents a molecular basis for C anticarcinogenic and anti-inflammatory activities [109]. Worth mentioning, the anti-inflammatory properties of *C. longa* and *Allium hookeri* have been shown to be principally mediated via the inhibition of NFKB and COX2 pathways [110]. These two pathways are of crucial importance also for the potential chemopreventive role of C, as demonstrated in epithelial colon cells [111].

Additional proinflammatory cytokines, namely CXCL2, CXCL3, CXCL5 (the most significantly affected gene), and CXCL8, were downregulated in the C + AFB1 group, most likely mitigating AFB1-induced toxic effects. To confirm this, 20 μM C suppressed CXCL5 gene expression in human middle ear epithelial cells after 1 h of exposure [112].

The upregulation of FNDC4 gene we observed goes in the same main stream of an overall anti-inflammatory response. This transcript, involved in cell adhesion, has been shown to be downregulated in BFH12 cells exposed to AFB1 [43], but it has recently been proven to act as an anti-inflammatory factor in mouse intestine and colon [113].

Scrolling through the list of DEGs, also the second most significantly downregulated gene is implicated in inflammatory processes. It is the colony-stimulating factor 3 (CSF3), playing a central role in inducing inflammation (e.g., in mice [114]).

Finally, the complement components CFB, C2, and C3, negatively modulated by C alone, were downregulated even in cell coincubated with C + AFB1, again suggesting a decreased inflammatory response to AFB1 attributable to the C treatment.

#### 4.4.3. Cancer

The functional analysis highlighted the significant enrichment of several terms connected to cancer and carcinogenic processes. The enrichment of the KEGG term “chemical carcinogenesis” was mainly represented by genes involved in the metabolism of carcinogens, such as GSTs (GSTA1, GSTA2, GSTM1, MGST1, and MGST3) and epoxide hydrolase 1 (EPHX1), involved in AFBO detoxification; these genes were all upregulated by C treatment. To corroborate our findings, the decrease of hepatic epoxide hydrolase and GST mRNA levels observed in broiler chicks given AFB1 was mitigated by *C. longa* inclusion in the AFB1 diet [33,39].

The enriched KEGG term “pathway in cancer” was represented by key genes promoting carcinogenesis. For instance, the signal transducer and activator of transcription 3 (STAT3), as well as STAT6, were both downregulated in C + AFB1 cells when compared to those exposed to AFB1 only. Both genes, albeit STAT6, to a minor extent, play a fundamental role in inhibiting the antitumor immunity [115]; indeed, they are involved in several signaling pathways controlling oncogenes. Furthermore, STAT3 is a well-known target of C (e.g., [116]). Likewise, mRNA levels of three oncogenes, namely ETS1, MDM2, and ARAF, were decreased by C treatment; on the contrary, the growth arrest and DNA-damage-inducible G (GADD45G), a stress-responsive gene involved in tumor suppression, was upregulated in C + AFB1 exposed cells. Overall, these findings strengthen the potential anticarcinogenic mechanism triggered by this flavonoid.

#### 4.4.4. Drug Metabolism and Drug Transporters

As expected, several genes involved in drug metabolism and transport were affected by C pretreatment, thus demonstrating how C most likely affects AFB1 uptake and biotransformation. Cattle CYP3A28 (an orthologue of human CYP3A4 and the main CYP3A isoform expressed in BFH12 cells: [117]) is a key player in AFB1 metabolism, being responsible for the synthesis of AFBO (bioactivation) [11]. In a previous study, AFB1 increased significantly CYP3A28 mRNA level [43], and in our experimental conditions such an induction was counteracted by C, with a resulting significant gene downregulation. These same effects have been observed also at the protein level, evaluating the CYP3A catalytic activity by P450-GloTM CYP3A4 assay with Luciferin-IPA as substrate. To the best of the authors’ knowledge, this approach has been previously used only once in cattle [118]. In our experimental conditions, it appeared sensitive enough to highlight the counteracting effect of C on CYP3A enzymatic activity in C + AFB1 and CL + AFB1 exposure conditions. To confirm our data, an inhibitory effect of curcuminoids upon CYP3A-dependent catalytic activity has been previously noticed in vitro and ex vivo in humans and rats [65,66,67,119].

A second CYP downregulated by C treatment was CYP26B1. This gene is a retinoic acid hydroxylase that metabolizes retinoic acid, and it is not considered as a classic xenobiotic metabolizing enzyme. Despite this, both the significance (FDR = 1.57 × 10^−7^) and the extent (log_2_FC = 4.36) of the observed changes in mRNA levels let us speculate that CYP26B1 might play an important role in BFH12 response to AFB1. Conversely, some other CYPs were induced by C treatment. For instance, CYP2B6 and CYP1A1 were both induced by C, albeit this latter one with a marginal significance (FDR = 0.066). In human liver, AFB1 has been reported to be a substrate of CYP2B6 [120]. Hence, it should be conceivable to hypothesize this CYP might contribute to AFB1 bioactivation in humans; however, its role in bovine liver remains unclear. Controversial results were obtained with CYP1A1; this gene was previously shown to be downregulated by AFB1 [43], and in the present study the treatment with C reverted this transcriptional variation. Nevertheless, C has been reported to inhibit some substrate-dependent CYP1A1 induction [121,122].

Several GSTs and EPHX1, as mentioned above, are induced by C. This result is consistent with the published literature (e.g., [31,33,37]) and further corroborates the protective role of C against AFB1 toxicity, which includes the activation of a series of detoxifying molecular mechanisms.

Interestingly, ABC transporters seem to play a role in reducing AFB1 toxicity. Except for ABCA1, ABCA12, and multidrug resistance protein 1 (MDR1), all downregulated, a total of seven genes encoding for ABC-transporters were upregulated by C treatment. Among them, the efflux transporter ABCG2 (also known as breast cancer resistance protein, BCRP), was the most significantly upregulated gene (log_2_FC = 1.43, FDR = 0.001). This is a controversial result, because curcuminoids are considered as ABCG2 inhibitors [123]. However, a possible interpretation of our result is that ABCG2 upregulation might mitigate AFB1 toxicity decreasing its cellular uptake, as previously demonstrated in MDCK-II cells transduced with ABCG2 [124]. Among the three downregulated drug transporters, MDR1 was the most significant one (log_2_FC = 1.52, FDR = 2 × 10^−4^). Alike ABCG2, MDR1 is recognized worldwide as an efflux drug transporter, not only modulating the xenobiotic bioavailability but also increasing drug resistance phenomena [125]. Moreover, it has been reported its ability to transport AFB1 and its glutathione conjugates in vitro [126]. Nonetheless, it seems unlikely for MDR1 being essential for the AFB1 detoxification process [127]. Interestingly, in BFH12 cells the MDR1 gene was induced by AFB1 [43], and C reverted the AFB1-dependent mRNA modulation, thus suggesting that C counteracts anyway the molecular effects of the mycotoxin.

## 5. Conclusions

To the best of our knowledge, this is the first study assessing the potential benefits of curcuminoids in AFB1-exposed bovine cells (i.e., fetal hepatocytes) by combining cytotoxicity, whole-transcriptomic signatures, and specific post-transcriptional confirmatory assays. Based on the present results and the previously published literature, the following conclusions can be drawn. (1) A treatment with curcuminoids reduced AFB1 cytotoxicity. (2) Curcumin treatment considerably affected the transcriptome of bovine hepatic cells exposed to AFB1. (3) The pathways mostly affected by C, and driving protective mechanisms against AFB1 toxicity, are related to antioxidant response, defense response and inflammation, cancer, drug metabolism, and drug transport. (4) Prospective molecular studies are envisaged to confirm the role played by specific pathways/genes in AFB1 mechanistic toxicology and, consequently, better characterize the protective role of curcuminoids. (5) In vivo studies are clearly needed to test the use of curcumin in feeding strategies, its bioavailability and its beneficial effects on aflatoxicosis.

## Supplementary Materials

The following are available online at https://www.mdpi.com/2076-3921/9/11/1059/s1, Figure S1: Biotransformation of AFB1 in BFH12 cells, Figure S2: Real-time PCR: transcriptional changes induced by C. Figure S3: Real-time PCR: transcriptional changes induced by CL, Figure S4: MDS plot, Figure S5: Correlation between C and CL treatments (alone or in combination with AFB1), Figure S6: Gene Set Enrichment Analysis: C + AFB1 vs. AFB1, Table S1: Real-time PCR assays, Table S2: Sequencing and mapping results, Table S3: Differential expression analysis, Table S4: Comparison between top-10 DEGs in C vs. CTRL and CL vs. CTRL, Table S5: GO over-representation analysis, Table S6: KEGG over-representation analysis, Table S7: Gene Set Enrichment Analysis (KEGG pathways), Table S8. Overall gene expression level of the discussed genes, File S1: R code.
