# Supplementary material for: Curcumin Mitigates AFB1-Induced Hepatic Toxicity by Triggering Cattle Antioxidant and Anti-inflammatory Pathways: A Whole Transcriptomic In Vitro Study
Source: Antioxidants (Basel). 2020 Oct 29;9(11):1059. doi: 10.3390/antiox9111059 (PMC7692341; doi:10.3390/antiox9111059)
Supplement: Supplementary file 1 [file antioxidants-09-01059-s001.zip › SupplementaryMaterial/Captions_revised_proof.docx]

**Figure S1.** **Biotransformation of AFB1 in BFH12 cells.** Concentrations (ng/mL) of AFM1 measured in the medium after 48 hrs (i.e. at the end of the experiment, T96 hrs) of exposure to 3.6 μM AFB1 alone or in combination with increasing concentrations of C and CL. Data are expressed as mean concentration ± standard deviation of five independent cell culture experiments. Different letters above error bars indicate significant differences (p ≤ 0.05) among groups (Tukey's post-hoc test). C=curcumin; CL=*Curcuma longa*; A=AFB1.

**Figure S2.** **Real-time PCR: transcriptional changes induced by C.** The one-way ANOVA, followed by a Dunnett’s multi-comparisons test, was carried out to compare the gene expression level of cells exposed to increasing doses of C (the lowest dose was used as control in the comparisons). The same statistical approach was adopted to investigate the effects of C treatment, separately: all the conditions were compared to the AFB1 condition. Data are expressed in fold change vs. CTRL condition (i.e., PCB126) ± standard deviation. C=curcumin; A=AFB1. *p ≤ 0.05; **p ≤ 0.01; ***p ≤ 0.001.

**Figure S3.** **Real-time PCR: transcriptional changes induced by CL.** The one-way ANOVA, followed by a Dunnett’s multi-comparisons test, was carried out to compare the gene expression level of cells exposed to increasing doses of CL (the lowest dose was used as control in the comparisons). The same statistical approach was adopted to investigate the effects of CL treatment, separately: all the conditions were compared to the AFB1 condition. Data are expressed in fold change vs. CTRL condition (i.e., PCB126) ± standard deviation. CL=*Curcuma longa*; A=AFB1. *p ≤ 0.05; **p ≤ 0.01; ***p ≤ 0.001.

**Figure S4. MDS plot.** The plot shows distances between expression profiles of the eighteen RNA-seq libraries evaluated in this study. Biological replicates are represented by b, c and d letters. C=curcumin; CL=*Curcuma longa*; A=AFB1.

**Figure S5. Correlation between C and CL treatments (alone or in combination with AFB1).** Pearson correlation between log_2_FC obtained by comparing C vs. CTRL and CL vs. CTRL (**a**), and C+AFB1 vs. AFB1 and CL+AFB1 vs. AFB1 (**b**). All the expressed genes were considered, even if not DEGs. The correlation coefficient (*r*) was calculated in R using the *cor* function (*stats* package), while the correlation plot was built using the *plot* function (*graphics* package). C=curcumin; CL=*Curcuma longa*.

**Figure S6. Gene Set Enrichment Analysis: C+AFB1 vs. AFB1.** The ridgeplot visualizes the expression distributions of core enriched genes for GSEA enriched KEGG pathways. Gradient colour reflects the adjusted p-values (Benjamini–Hochberg method).

**Table S1.** **Real-time PCR assays**. The table reports the complete sequence of forward (F) and reverse (R) primers, the Ensembl gene ID of each target gene, and information about the qPCR assays (efficiency and dynamic range).

**Table S2. Sequencing and mapping results.** The table reports the RNA-seq libraries sequenced including for each of them i) the number of raw reads obtained, ii) the number of reads after trimming and rRNAs removal iii) the number of mapped reads (and the percentage of mapped reads).

**Table S3. Differential expression analysis.** The table reports the EdgeR glmTREAT output for the tests performed (“C vs. CTRL”; “CL vs. CTRL”; “C+AFB1 vs. AFB1”; “CL+AFB1 v.s AFB1”). Ensembl gene description, gene name, log2 fold change (logFC), mean log count per millions (logCPM), nominal p-value and false discovery rate (FDR) are reported for all the genes evaluated. The transcriptional changes (i.e. upregulation, downregulation, or not significant) induced by antioxidants were also specified for all the genes.

**Table S4. Comparison between top-10 DEGs in C vs. CTRL and CL vs. CTRL.** Nine out of the top-10 up- and downregulated genes shared between the two analyses. Log_2_FC resulted from the two analyses were also reported.

**Table S5. GO over-representation analysis.** Tables report the over-represented GO terms in the list of DEGs resulted from pair-wise tests conducted in this study. Terms highlighted in grey are those resulting after redundancy removal.

**Table S6. KEGG over-representation analysist.** Tables report the over-represented KEGG terms in the list of DEGs resulted from the pair-wise comparisons C vs. CTRL and C+AFB1 vs. AFB1.

**Table S7. Gene Set Enrichment Analysis (KEGG pathways).** Tables report the enriched KEGG terms in the comparison C vs. CTRL and C+AFB1 vs. AFB1.

**Table S8. Overall gene expression level of the discussed genes.** Mean normalized counts per million reads mapped (CPM) for each experimental group. The red color gradient highlights the expression level of each gene across the different experimental groups. It has been also reported if these genes were significantly up or downregulated by cucruminoids used alone or in combination with AFB1.

**File S1.** **R code.** Complete R code used for the differential gene expression analysis (edgeR), the data visualization, and the enrichment analysis.
