# Supplementary material for: Curcumin Mitigates AFB1-Induced Hepatic Toxicity by Triggering Cattle Antioxidant and Anti-inflammatory Pathways: A Whole Transcriptomic In Vitro Study
Source: Antioxidants (Basel). 2020 Oct 29;9(11):1059. doi: 10.3390/antiox9111059 (PMC7692341; doi:10.3390/antiox9111059)
Supplement: Supplementary file 1 [file antioxidants-09-01059-s001.zip › SupplementaryMaterial/FileS1_revised.docx]

**File S1. R code.** Complete R code used for the differential gene expression analysis (edgeR), the data visualization, and the enrichment analysis.

- *DIFFERENTIAL EXPRESSION ANALYSIS (EdgeR)*

*Read data and set groups*

>x <- read.delim("input.txt",row.names="Gene", stringsAsFactors=FALSE, header = TRUE)

>targets<-readTargets(file="targets.txt")

>Group <- factor(paste(targets$Condition))

>cbind(targets,Group=Group)

*Build the generalized linear model that will be used for differential expression testing*

>dge <- DGEList(counts=x, group=Group)

>keep <- filterByExpr(dge)

>table(keep)

>dge <- dge[keep, , keep.lib.sizes=FALSE]

>cpm <- cpm(dge)

>lcpm <- cpm(dge, log=TRUE)

*Normalization*

>dge_norm <- calcNormFactors(dge)

*MDS plot*

>par(mfrow=c(1,1))

>col.cell<-c("blue","blue","blue","orange","orange","orange","brown","brown","brown","green",

+ "green","green","darkgreen","darkgreen","darkgreen","black","black","black")

>plotMDS(dge_norm, col=col.cell)

>title(main="Plot MDS")

*Set study design*

design <- model.matrix(~0+Group, data=dge_norm$samples)

>colnames(design)<-levels(dge_norm$samples$group)

*Estimate dispersion*

>dge_Disp <- estimateDisp(dge_norm, design, robust=TRUE)

*Set contrasts*

>my.contrasts<-makeContrasts(C=C-PCB126,

+ CL=CL-PCB126,

+ C_AFLA=C_AFB1-AFB1,

+ CL_AFLA=CL_AFB1-AFB1, levels=design)

*Quasi-likelihood F-tests*

>fit <- glmQLFit (dge_Disp, design, robust=TRUE)

>TREAT.C <- glmTreat (fit, contrast=my.contrasts[,"C"], lfc=log2(1.5))

> Res_TreatQLF_C <- topTags (TREAT.C, n=Inf, adjust.method="fdr", srt.by="PValue")

>write.table (file="QLF_TREAT_C.txt", Res_TreatQLF_C, sep='\t', quote=F, row.names=T)

>is.de <- decideTestsDGE (TREAT.C)

>summary (is.de)

>TREAT.CL<- glmTreat (fit, contrast=my.contrasts[,"CL"], lfc=log2(1.5))

>Res_TreatQLF_CL<- topTags (TREAT.CL, n=Inf, adjust.method="fdr", sort.by="PValue")

>write.table (file="QLF_TREAT_CL.txt", Res_TreatQLF_CL, sep='\t', quote=F,row.names=T)

>is.de <- decideTestsDGE (TREAT.CL)

>summary (is.de)

>TREAT.C_AFLA <- glmTreat (fit, contrast=my.contrasts[,"C_AFLA"], lfc=log2(1.5))

>Res_TreatQLF_C_AFLA <- topTags (TREAT.C_AFLA, n=Inf, adjust.method="fdr", sort.by="PValue")

>write.table (file="QLF_TREAT_C_AFLA.txt", Res_TreatQLF_C_AFLA, sep='\t', quote=F,row.names=T)

>is.de <- decideTestsDGE (TREAT.C_AFLA)

>summary (is.de)

>TREAT.CL_AFLA <- glmTreat (fit, contrast=my.contrasts[,"CL_AFLA"], lfc=log2(1.5))

>Res_TreatQLF_CL_AFLA <- topTags (TREAT.CL_AFLA, n=Inf, adjust.method="fdr", sort.by="PValue")

>write.table (file="QLF_TREAT_CL_AFLA.txt", Res_TreatQLF_CL_AFLA, sep='\t', quote=F, row.names=T)

>is.de <- decideTestsDGE (TREAT.CL_AFLA)

>summary (is.de)

- *PEARSON CORRELATION*

>C=TREAT.C$table$logFC

>CL=TREAT.CL$table$logFC

>cor(C,CL,use="all.obs", method=c("pearson"))

>plot(C,CL,xlab="logFC C",ylab="logFC CL")

- *FUNCTIONAL ANALYSIS*

>library (clusterProfiler)

>library (enrichplot)

>library (org.Bt.eg.db)

1. ***Transcriptional effects of curcuminoids (identical for C or CL)***

*1.1 Data input (DEGs and logFC)*

>C <- read.csv("DEGS_C.csv", sep=";")

>geneList <- C [,2]

>names(geneList) <- as.character(C[,1])

>geneList <- sort(geneList, decreasing = TRUE)

>DEGs <- names(geneList)

*1.2 Set a background (expressed genes)*

>back <- topTags(TREAT.C, n = Inf)$table

>back_list<- row.names(back)

*1.3 GO over-representation test*

>enrichBP_DEGS_C <- enrichGO (gene = DEGs, OrgDb = org.Bt.eg.db, keyType = "ENSEMBL", ont = "BP", pvalueCutoff = 0.05, pAdjustMethod = "BH", universe = back_list, qvalueCutoff = 0.2, minGSSize = 2, maxGSSize = 500, readable = TRUE, pool = FALSE)

>write.table(file="enrichBP_DEGS_C.txt",enrichBP_DEGS_C, sep='\t', quote=F,row.names=T)

>enrichBP_DEGS_C_simply <- simplify(enrichBP_DEGS_C, cutoff=0.5, by="p.adjust", select_fun=min)

>write.table(file="enrichBP_DEGS_C_simply.txt",enrichBP_DEGS_C_simply, sep='\t', quote=F,row.names=T)

#Dotplot

>dotplot(enrichBP_DEGS_C_simply, showCategory=10)

#Gene-concept Network

>enrichBP_DEGS_C_GeneNames <- setReadable(enrichBP_DEGS_C_simply, "org.Bt.eg.db", keyType="ENSEMBL")

>enrichBP_DEGS_C_GeneNames

>p1 <- cnetplot(enrichBP_DEGS_C_GeneNames, foldChange=geneList, circular = FALSE, colorEdge = TRUE)

>plot_grid(p1)

1. ***Effects of curcuminoids on AFB1-induced transcriptional changes (identical for C or CL)***

*2.1 Data input (DEGs and logFC)*

>C_A <- read.csv("DEGS_C_A.csv", sep=";")

>geneList <- C_A [,2]

>names(geneList) <- as.character(C_A[,1])

>geneList <- sort(geneList, decreasing = TRUE)

>DEGs <- names(geneList)

*2.2 Set a background (expressed genes)*

>back <- topTags(TREAT.C_AFLA, n = Inf)$table

>back_list<- row.names(back)

*2.3 GO over-representation test*

>enrichBP_DEGS_C_A <- enrichGO (gene = DEGs, OrgDb = org.Bt.eg.db, keyType = "ENSEMBL", ont = "BP", pvalueCutoff = 0.05, pAdjustMethod = "BH", universe = back_list, qvalueCutoff = 0.2, minGSSize = 2, maxGSSize = 500, readable = TRUE, pool = FALSE)

>write.table(file="enrichBP_DEGS_C_A.txt",enrichBP_DEGS_C_A, sep='\t', quote=F,row.names=T)

>enrichBP_DEGS_C_A_simply <- simplify(enrichBP_DEGS_C_A, cutoff=0.5, by="p.adjust", select_fun=min)

>write.table(file="enrichBP_DEGS_C_A_simply.txt",enrichBP_DEGS_C_red, sep='\t', quote=F,row.names=T)

*2.4 Convert ENSEMBL gene IDs to gene names*

>enrichBP_Gene_DEGS_C_A_simply <- setReadable(enrichBP_DEGS_C_A_simply, "org.Bt.eg.db", keyType="ENSEMBL")

>enrichBP_Gene_DEGS_C_A_simply

#Gene-concept Network

>p <- cnetplot(enrichBP_Gene_DEGS_C_A_simply, categorySize="pvalue", foldChange=geneList, colorEdge = TRUE)

>plot_grid(p)

*2.5 Convert ENSEMBL IDs to ENTREZ IDs*

>DEGs_ENTREZ<- bitr(DEGs, fromType = "ENSEMBL", toType = c("ENTREZID"), OrgDb = org.Bt.eg.db)

>DEGs_ENTREZ_OK<-DEGs_ENTREZ$ENTREZ

>back_ENTREZ<- bitr(back_list, fromType = "ENSEMBL", toType = c("ENTREZID"), OrgDb = org.Bt.eg.db)

>Back_ENTREZ_OK<-back_ENTREZ$ENTREZID

*2.6 KEGG over-representation test*

>KEGG_DEGS_C_A <- enrichKEGG (gene = DEGs_ENTREZ_OK, organism ="bta", pvalueCutoff = 0.05, pAdjustMethod = "BH", universe = Back_ENTREZ_OK, minGSSize = 5, maxGSSize = 500, use_internal_data = FALSE)

#Dotplot

>dotplot(KEGG_DEGS_C_A, showCategory=20)

*2.7 KEGG Gene Set Enrichment Analysis*

>inputGSEA_C_A <- read.csv("input_GSEA_C_A.csv", sep=";")

>geneList_GSEA <- inputGSEA_C_A [,2]

>names(geneList_GSEA) <- as.character(inputGSEA_C_A[,1])

>geneList_GSEA <- sort(geneList_GSEA, decreasing = TRUE)

>kegg <- gseKEGG(geneList = geneList_GSEA,

organism = "bta",

minGSSize = 3,

maxGSSize = 800,

pvalueCutoff = 0.05,

pAdjustMethod = "BH",

keyType = "ncbi-geneid")

write.table(file="gseaKEGG_C_A.txt",kegg, sep='\t', quote=F,row.names=T)

#Ridgeplot

>ridgeplot(kegg) + labs(x = "enrichment distribution")
