# Supplementary material for: Curcumin Mitigates AFB1-Induced Hepatic Toxicity by Triggering Cattle Antioxidant and Anti-inflammatory Pathways: A Whole Transcriptomic In Vitro Study
Source: Antioxidants (Basel). 2020 Oct 29;9(11):1059. doi: 10.3390/antiox9111059 (PMC7692341; doi:10.3390/antiox9111059)
Supplement: Supplementary file 1 [file antioxidants-09-01059-s001.zip › SupplementaryMaterial/TableS1.docx]

**Table S1. Real-time PCR assays.** The table reports the complete sequence of forward (F) and reverse (R) primers, the Ensembl gene ID of each target gene, and information about the qPCR assays (efficiency and dynamic range).

| **Gene** | **Primer sequence (5’-3’)** | **Ensembl Gene ID** | **Efficiency (%)** | **Dynamic range (C_P_)** |
| --- | --- | --- | --- | --- |
| AHR | F: GTGCAGAAAACTGTCAAGCC  R: GCAACATCAAAGAAGCTCTTG | ENSBTAG00000007746 | 96.40 | 20.96–31.72 |
| ARNT | F: TTTCCTCACTGATCAGGAAC  R: TCCAGGATACGCCCTGTC | ENSBTAG00000021037 | 100.20 | 19.63–30.04 |
| CAT | F: CTCACTTTGACCGGGAGAGA  R: TGACCTCAAAGTAGCCAAAAGC | ENSBTAG00000020980 | 96.00 | 19.23–30.77 |
| CYP1A1 | F: GACAGCAATCTGAACGGCTTTTA  R: CCAGAGCTTCTGGTCATGGT | ENSBTAG00000001021 | 97.20 | 19.87–31.22 |
| CYP1B1 | F: CACCAGGTATTCGGAAGTGC  R: AAGAAAGGCCATGACGTAGG | ENSBTAG00000010531 | 107.20 | 19.93–30.32 |
| CYP3A28 | F: CCCCTTGAAAATAAGCAGTCA  R: ATCAAGCCCCCTGAAATTCT | ENSBTAG00000052665 | 101.30 | 24.16–32.82 |
| GPX1 | F: GGGCATCAGGAAAACGCC  R: GCATAAAGTTGGGCTCGAACC | ENSBTAG00000054195 | 98.40 | 18.46–29.73 |
| KEAP1 | F: GAGCAGATTGGCTGTACGGA  R: GCAGTGGGACAGGTTGAAGA | ENSBTAG00000003543 | 103.40 | 19.23–30.22 |
| NQO1 | F: CGGAATAAGAAGGCAGTGCT  R: AGCCACAGAAGTGCAGAGTG | ENSBTAG00000020632 | 109.60 | 23.98–34.86 |
| NRF2 | F: AGCTTTTGGCAGAGACATTCC  R: TGGGCTCTCGATGTGGCT | ENSBTAG00000019255 | 99.70 | 17.78–28.96 |
| SOD2 | F: GTGAACAACCTCAACGTCGC  R: CTTCAACGCAGGCTGCAGAG | ENSBTAG00000006523 | 96.10 | 17.77–31.92 |
| SOD1 | F: ATCCACTTCGAGGCAAAGGG  R: CTCCAAACTGATGGACGTGGA | ENSBTAG00000018854 | 109.60 | 16.52–29.77 |
| RPLP0 | F: CAACCCTGAAGTGCTTGACAT  R: AGGCAGATGGATCAGCCA | ENSBTAG00000017389 | 99.10 | 14.21–30.20 |
| TBP | F: ACAACAGCCTCCCACCCTATGC  R: GTGGAGTCAGTCCTGTGCCGTAA | ENSBTAG00000007686 | 93.00 | 21.26–35.92 |
