# Supplementary material for: Curcumin Mitigates AFB1-Induced Hepatic Toxicity by Triggering Cattle Antioxidant and Anti-inflammatory Pathways: A Whole Transcriptomic In Vitro Study
Source: Antioxidants (Basel). 2020 Oct 29;9(11):1059. doi: 10.3390/antiox9111059 (PMC7692341; doi:10.3390/antiox9111059)
Supplement: Supplementary file 1 [file antioxidants-09-01059-s001.zip › SupplementaryMaterial/TableS2.docx]

**Table S2. Sequencing and mapping results.** The table reports the RNA-seq libraries sequenced, including for each of them: i) the number of raw reads obtained; ii) the number of reads after trimming and rRNAs removal; iii) the number of mapped reads (and the percentage of mapped reads).

| Sample ID | N° raw reads | N° reads after trimming and rRNAs removal | N° of reads mapping (%) |
| --- | --- | --- | --- |
| PCB126_B | 22,928,709 | 22,823,199 | 22,628,132 (99.15) |
| PCB126_C | 25,807,296 | 25,653,873 | 25,436,649 (99.15) |
| PCB126_D | 43,005,307 | 42,793,203 | 42,432,959 (99.16) |
| AFB1_B | 20,399,647 | 20,286,542 | 20,106,412 (99.11) |
| AFB1_C | 23,877,815 | 23,632,276 | 23,428,800 (99.14) |
| AFB1_D | 10,774,628 | 10,714,111 | 10,614,084 (99.07) |
| CL_B | 31,180,310 | 30,977,159 | 30,699,454 (99.10) |
| CL_C | 24,746,080 | 24,575,650 | 24,345,413 (99.06) |
| CL_D | 30,253,060 | 30,022,170 | 29,730,267 (99.03) |
| C_B | 33,483,977 | 33,284,648 | 32,934,585 (98.95) |
| C_C | 18,538,819 | 18,413,707 | 18,247,107 (99.10) |
| C_D | 26,142,970 | 25,943,458 | 25,699,384 (99.06) |
| CL+AFB1_B | 36,126,726 | 35,974,571 | 35,667,868 (99.15) |
| CL+AFB1_C | 33,415,173 | 33,199,825 | 32,848,973 (98.94) |
| CL+AFB1_D | 40,281,413 | 40,043,950 | 39,663,688 (99.05) |
| C+AFB1_B | 38,456,411 | 38,281,201 | 37,937,937 (99.10) |
| C+AFB1_C | 33,859,220 | 33,700,198 | 33,389,131 (99.08) |
| C+AFB1_D | 20,310,082 | 20,148,011 | 19,939,963 (98.97) |
