# Supplementary figures and images for: Curcumin Mitigates AFB1-Induced Hepatic Toxicity by Triggering Cattle Antioxidant and Anti-inflammatory Pathways: A Whole Transcriptomic In Vitro Study
Source: Antioxidants (Basel). 2020 Oct 29;9(11):1059. doi: 10.3390/antiox9111059 (PMC7692341; doi:10.3390/antiox9111059)

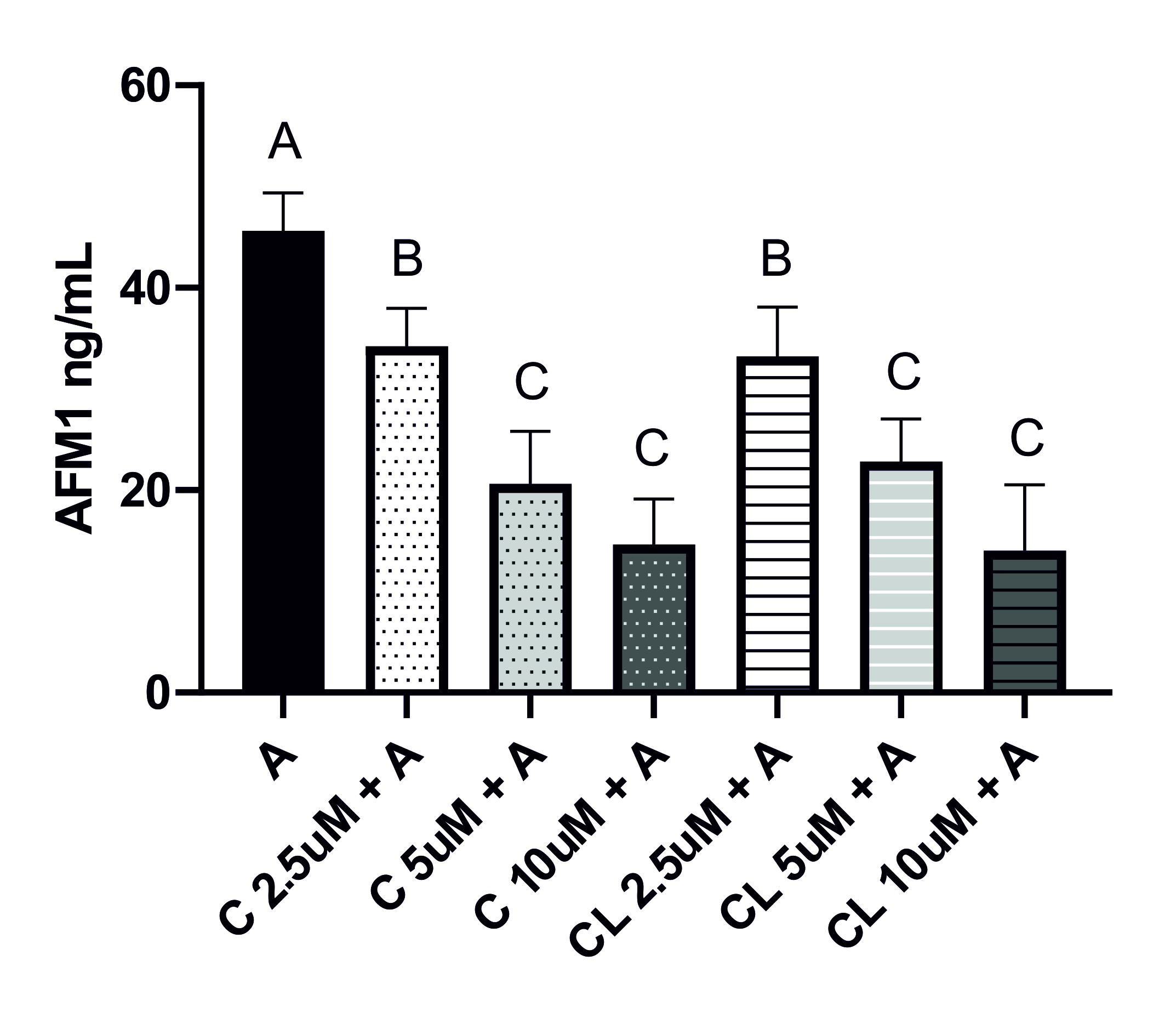

Supplement: Supplementary file 1 [file antioxidants-09-01059-s001.zip › SupplementaryMaterial/FigureS1.tiff]

CYP1A1

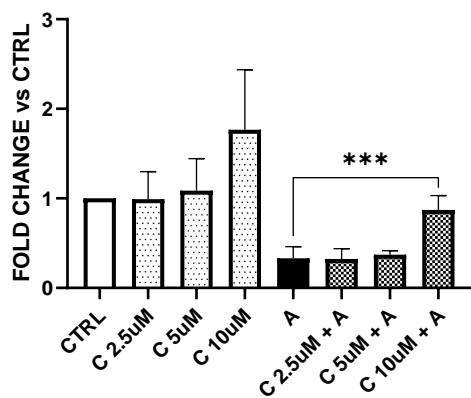

CYP1B1

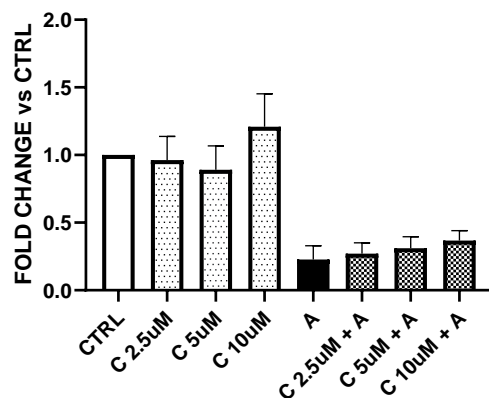

CYP3A28

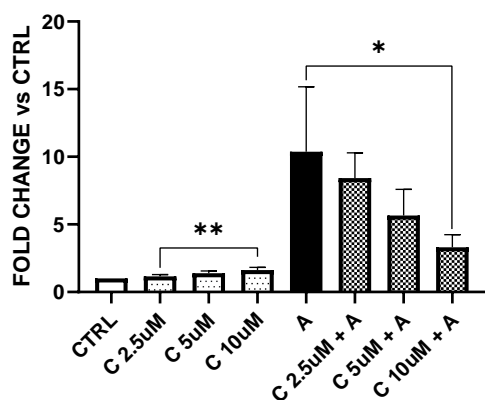

NQO1

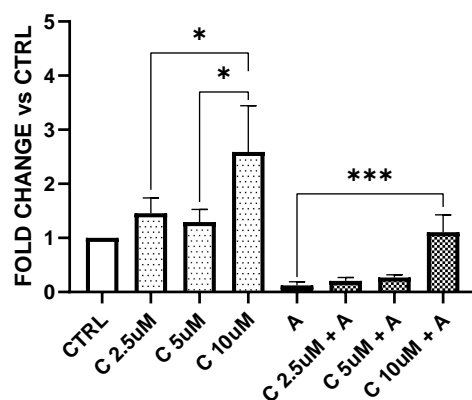

AHR

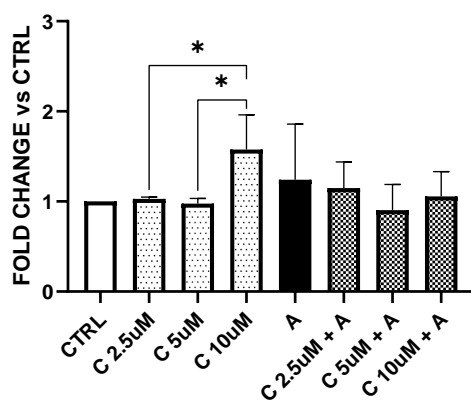

ARNT

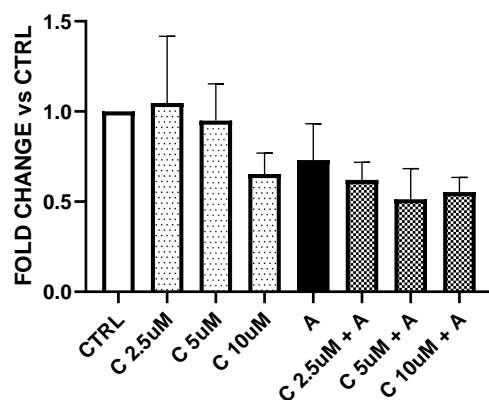

NRF2

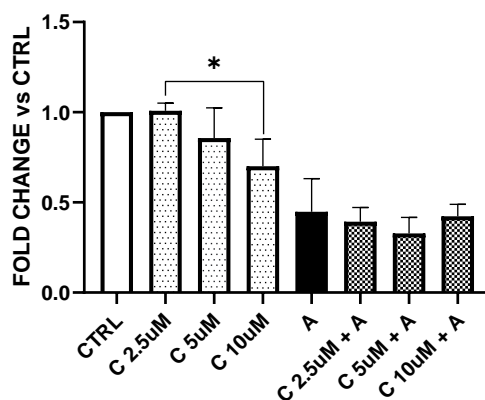

KEAP1

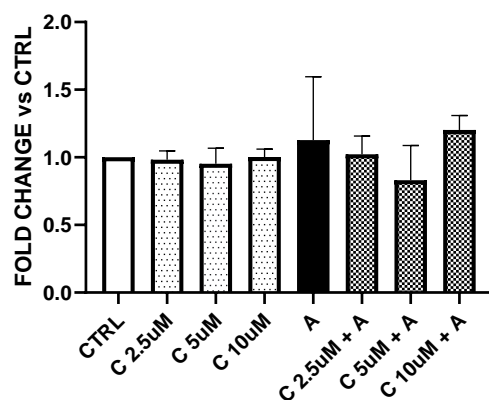

CAT

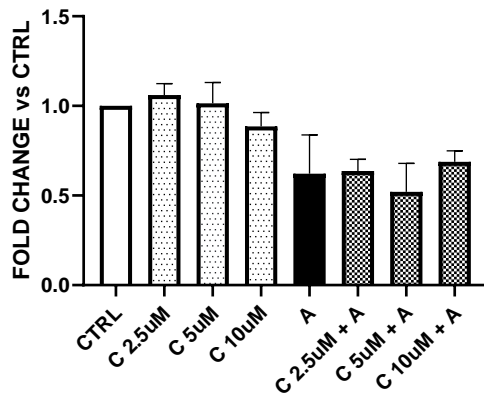

GPX1

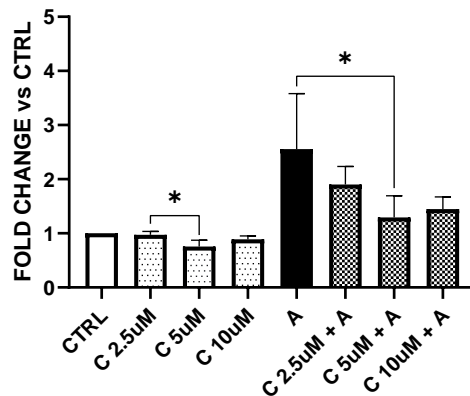

SOD1

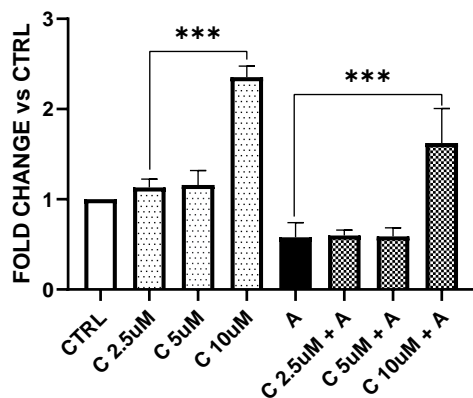

SOD2

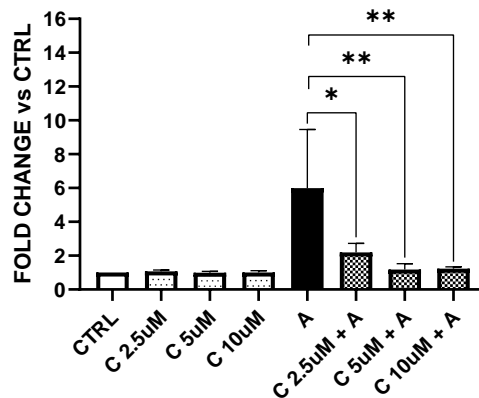

Supplement: Supplementary file 1 [file antioxidants-09-01059-s001.zip › SupplementaryMaterial/FigureS2.pdf]

CYP1A1

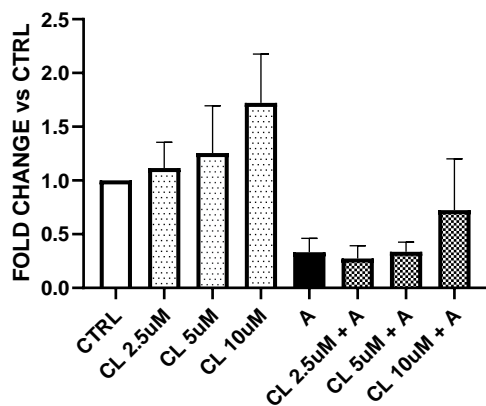

CYP1B1

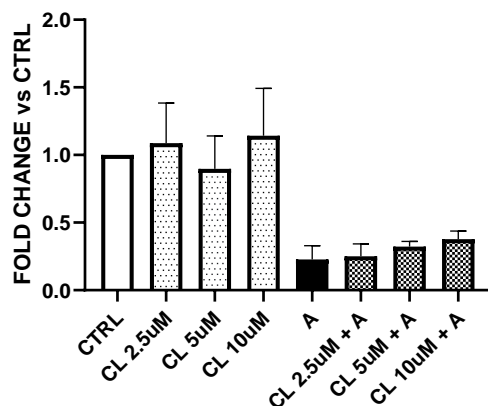

CYP3A28

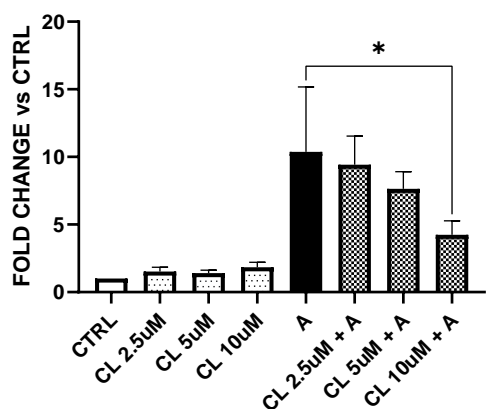

NQO1

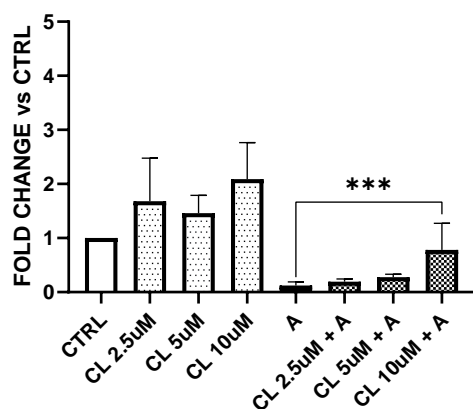

AHR

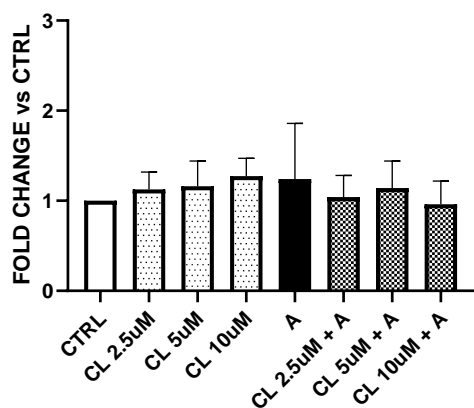

ARNT

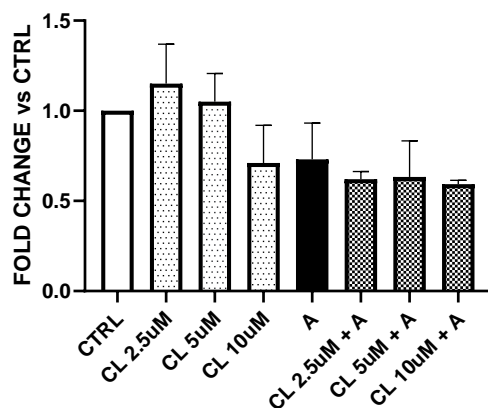

NRF2

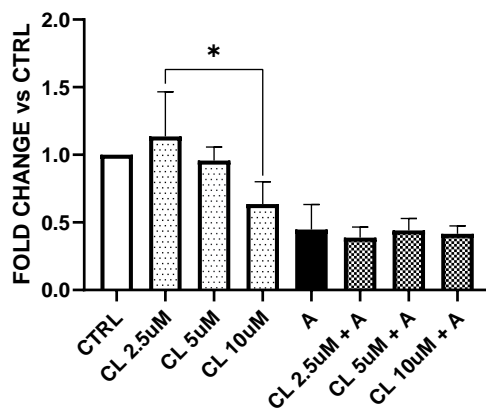

KEAP1

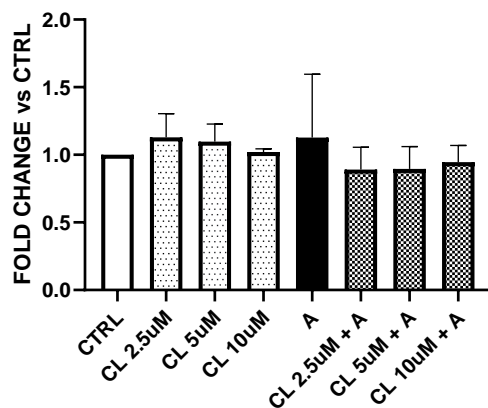

CAT

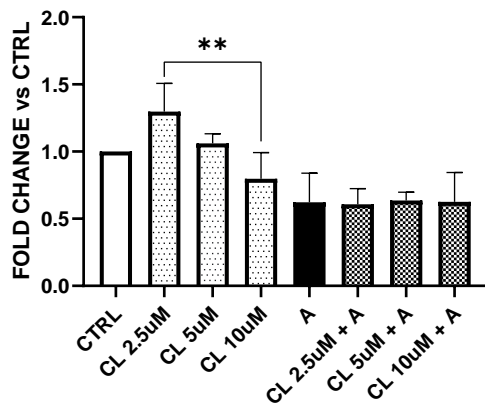

GPX1

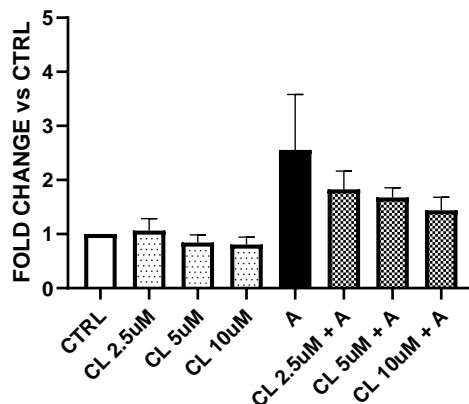

SOD1

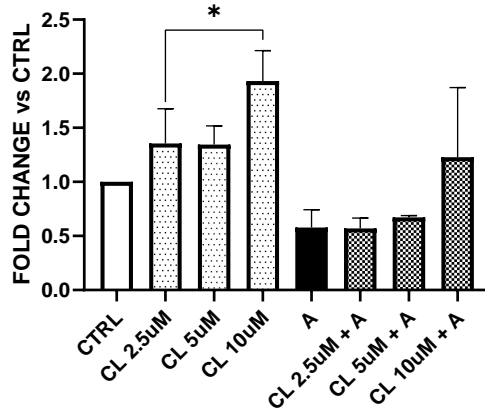

SOD2

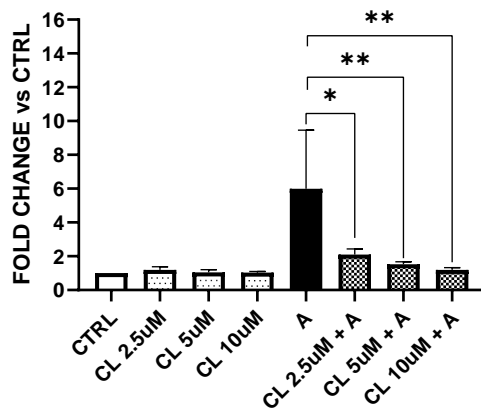

Supplement: Supplementary file 1 [file antioxidants-09-01059-s001.zip › SupplementaryMaterial/FigureS3.pdf]

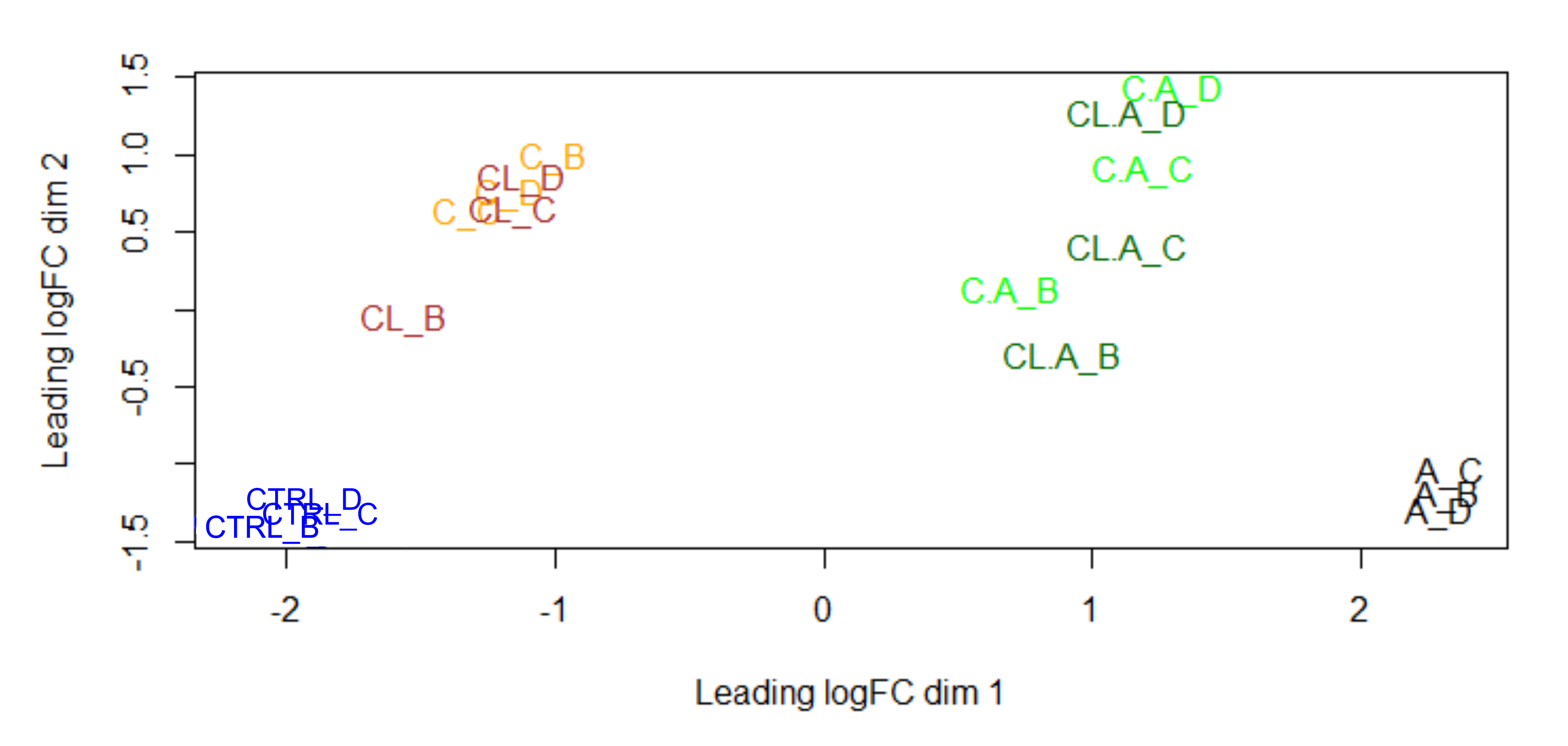

Supplement: Supplementary file 1 [file antioxidants-09-01059-s001.zip › SupplementaryMaterial/FigureS4_revised.tiff]

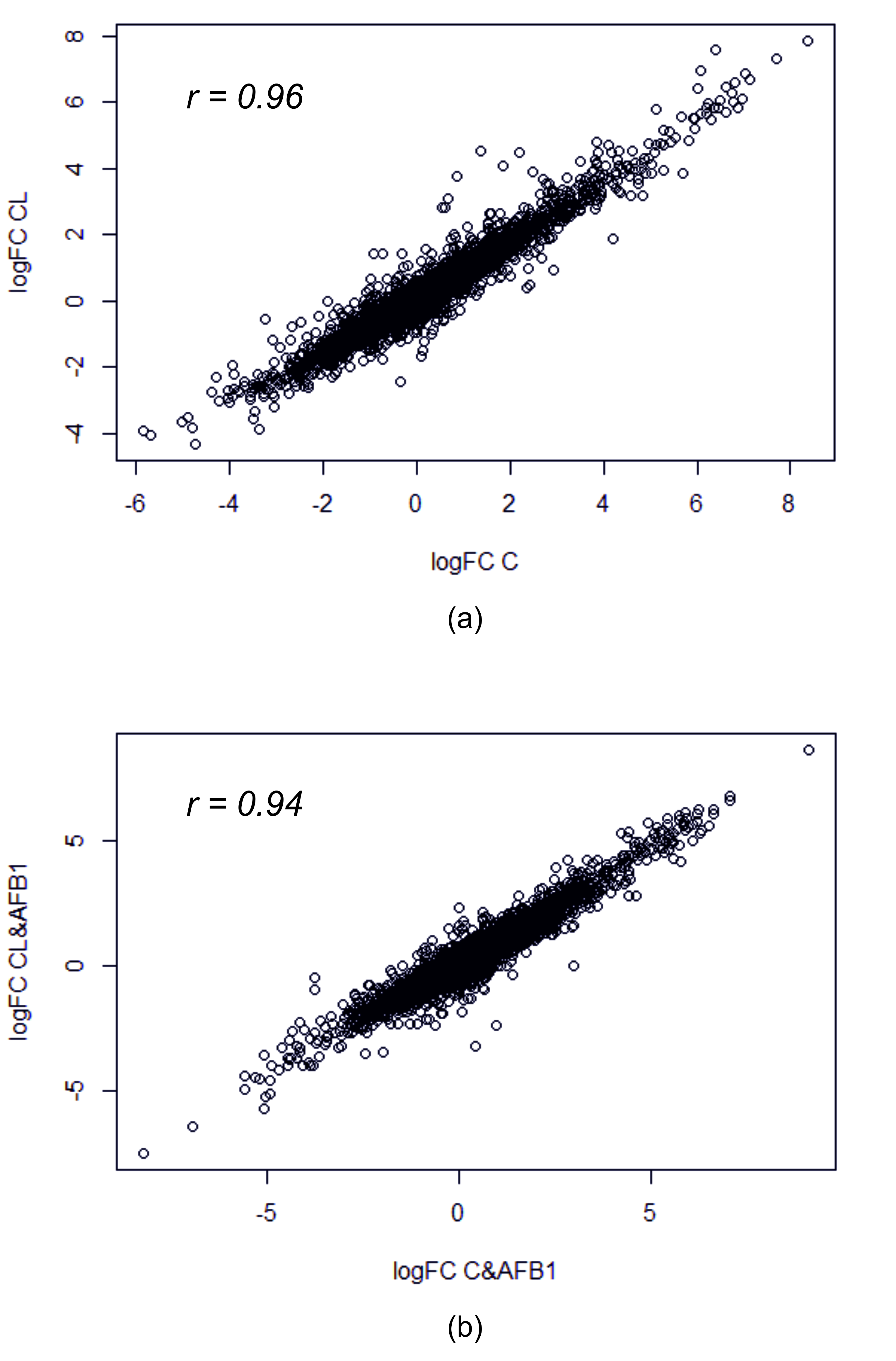

Supplement: Supplementary file 1 [file antioxidants-09-01059-s001.zip › SupplementaryMaterial/FigureS5.tiff]

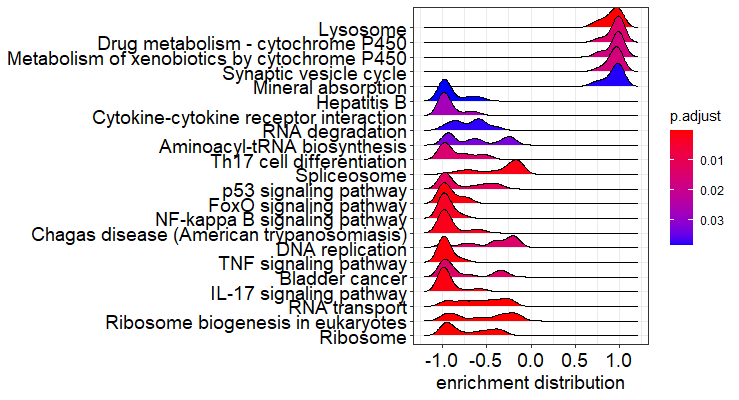

Supplement: Supplementary file 1 [file antioxidants-09-01059-s001.zip › SupplementaryMaterial/FigureS6.tiff]
